# Food intake biomarkers for berries and grapes

**DOI:** 10.1186/s12263-020-00675-z

**Published:** 2020-09-23

**Authors:** M. Ulaszewska, M. Garcia-Aloy, N. Vázquez-Manjarrez, M. T. Soria-Florido, R. Llorach, F. Mattivi, C. Manach

**Affiliations:** 1 Fondazione Edmund Mach, Research and Innovation Centre Food Quality and Nutrition, Via Mach 1, 38010 San Michele all’Adige, Italy; 2 Center for Omics Sciences, Proteomics and Metabolomics Facility – ProMeFa, IRCCS San Raffaele Scientific Institute, Milan, Italy; 3 Biomarkers and Nutrimetabolomic Laboratory, Department of Nutrition, Food Sciences and Gastronomy, Food Technology Reference Net (XaRTA), Nutrition and Food Safety Research Institute (INSA-UB), Faculty of Pharmacy and Food Sciences, University of Barcelona, Barcelona, Spain; 4 CIBER de Fragilidad y Envejecimiento Saludable (CIBERFES), Instituto de Salud Carlos III, Barcelona, Spain; 5 Université Clermont Auvergne, INRAE, UNH, F-63000, Clermont–Ferrand, France; 6 Department of Nutrition, Exercise and Sports, University of Copenhagen, Copenhagen, Denmark; 7 Dirección de Nutrición, Instituto Nacional de Ciencias Médicas y Nutrición Slavador Zubiran, Mexico City, Mexico; 8 Department of Cellular, Computational and Integrative Biology, CIBIO, University of Trent, Trento, Italy

**Keywords:** Grape, Raisin, Strawberry, Blueberry, Blackberry, Cranberry, Raspberry, Blackcurrant, Biomarkers, Intake

## Abstract

Grapes and berries are two types of widely consumed fruits characterized by a high content in different phytochemicals. However, their accurate dietary assessment is particularly arduous, because of the already wide recognized bias associated with self-reporting methods, combined with the large range of species and cultivars and the fact that these fruits are popularly consumed not only in fresh and frozen forms but also as processed and derived products, including dried and canned fruits, beverages, jams, and jellies. Reporting precise type and/or quantity of grape and berries in FFQ or diaries can obviously be affected by errors. Recently, biomarkers of food intake (BFIs) rose as a promising tool to provide accurate information indicating consumption of certain food items. Protocols for performing systematic reviews in this field, as well as for assessing the validity of candidate BFIs have been developed within the Food Biomarker Alliance (FoodBAll) Project. This paper aims to evaluate the putative BIFs for blueberries, strawberries, raspberries, blackberries, cranberries, blackcurrant, and grapes. Candidate BFIs for grapes were resveratrol metabolites and tartaric acid. The metabolites considered as putative BFI for berries consumption were mostly anthocyanins derivatives together with several metabolites of ellagitannins and some aroma compounds. However, identification of BFIs for single berry types encountered more difficulties. In the absence of highly specific metabolites reported to date, we suggested some multi-metabolite panels that may be further investigated as putative biomarkers for some berry fruits.

## Introduction

Fruits are important components of a healthy diet [1]. Increased consumption of fruits and vegetables is recommended in dietary guidelines worldwide, and the intake of fruits like berries, which are rich in nutrients, vitamins, minerals, and phytochemicals, may play a role in disease prevention [116]. In terms of production worldwide, according to FAOSTAT (http://www.fao.org/faostat/en/# consulted on 28.02.2020), strawberries were the most intensively produced berry, dominated by China, USA, Mexico, and Turkey with production in kilotonnes (Kt): 2955.4 Kt, 1296.3 Kt, 653.6 Kt, and 440.9 Kt, respectively. Cranberries were the second in the ranking with USA and Canada producing 404.9 Kt and 195.2 Kt, respectively. Production of raspberries was dominated by Russia, Mexico, Serbia, and Poland with 165.8 Kt, 130.2 Kt, 127.0 Kt, and 115.6 Kt, respectively. The top three producers of grapes worldwide in 2018 were China, Italy and USA with the following kilotonnes: 13,397.0 Kt, 8513.6 Kt, and 6890.9 Kt. According to the European food consumption database, northern countries, especially Finland and Latvia but also Spain and Romani are the biggest consumers of berries within Europe, consuming 1.38 kg/year, 1.24 kg/year, 1.28 kg/year, and 1.17 kg/year. The strawberries (4.77 g/day) are consumed most frequently, followed by blueberries (1.34 g/day) [42]. Berry fruits are popularly consumed not only in fresh and frozen forms but also as processed and derived products, including dried and canned fruits, yogurts, beverages, jams, and jellies, making the evaluation of their intake rather delicate when using questionnaires. On the other hand, grapes, excluding wine, are mainly consumed as fresh fruits, and mean consumption in European countries, according to comprehensive European food consumption database, is 4.93 g/day [42]. Among them, Hungary and Romania consume the highest amount of grapes 9.7 g/day and 8.8 g/day, respectively [42, 180].

Berry fruits have attracted considerable attention due to their potential benefits to human health. Botanical classification defines berry as a fruit with seeds produced from the ovary of a single flower. According to this definition category, berries include blueberries, cranberries, lingonberries, *Ribes* species, grapes, but also bananas, tomatoes or eggplants. On the other hand, strawberries, raspberries, and blackberries are not botanical berries, however enter this category in common nomenclature. This review includes both botanical and commonly called berries: strawberries (*Fragaria × ananassa*), raspberries (*Rubus idaeus*) and blackberries (*Rubus fruticosus*, other *Rubus spp*.), blueberries (*Vaccinium* fruits*,* i.e, *V. myrtillus or V. corymbosum*), American cranberries (*Vaccinium macrocarpon*), and blackcurrant (*Ribes nigrum*). They provide high levels of antioxidants, vitamins, minerals, and fibres and are amongst the richest sources of phenolic compounds in the human diet [10, 43, 120]. The biological activities of berries have been mainly attributed to their high content of a diverse range of flavonoids (anthocyanins, flavonols, and flavanols), tannins (proanthocyanidins, ellagitannins, and gallotannins), phenolic acids (hydroxybenzoic and hydroxycinnamic acid derivatives), and lignans. In particular, cranberries are known for their effect on urinary tract infections, while blueberries (a proanthocyanidin-rich fruit) and strawberries (an ellagitannin-rich fruit) are actively studied for their impact on neuronal function and behavior [8, 152, 154, 159].

Grape also contains a wide variety of polyphenols with demonstrated cardioprotective properties. Their polyphenolic content is higher than in other fruits, in the range of 50–490 mg/100 g of fresh matter [18]. However, an important percentage of these are contained in the seeds [102]. Several studies have been focused on the polyphenolic composition of this food revealing its complexity, mainly consisting of proanthocyanidins, anthocyanins, stilbenes, flavan-3-ols, and phenolic acids and complex condensation structures [21, 170]. It has been demonstrated that polyphenolic compounds from red grapes could improve endothelial function in patients with coronary heart disease [93], age-related cognitive and motor function [90], or decreases in oxidative stress and inflammatory markers [24, 118]. Considering both the health benefits of grape consumption [180] and the metabolic fate of their components, it became interesting to be able to monitor its intake with an accurate measurement tool.

This work aimed to provide an overview of the metabolites found in biological fluids that could potentially act as BFIs for grape and different berry types, further evaluating their validity considering the current available information and identifying the aspects that require further investigation. There are several examples where a single metabolite could be a biomarker of a particular food intake, such as phloretin for apple intake [166]. However, the composition of each plant food is very complex (from the quantitative and qualitative point of view) and many of the compounds are widely distributed among a variety of foods [62]. Several polyphenols can be considered semi-ubiquitary, they are present in a wide range of plant foods, e.g. chlorogenic acids (in coffee and apple and other fruits) or flavan-3-ols (in cocoa, tea fruit, and wine), and thus, using a single-biomarker strategy is very often not appropriate. On the other hand, the intake of different foods may give rise to similar metabolites in biofluids losing their specificity for a certain dietary source [57]. The ellagitannins and procyanidins represent such cases. Ellagitannins are present in foods such as walnuts, strawberries, Rubus, and pomegranate and are largely metabolized by the microbiota, producing urolithin derivatives. Finally, urolithins indicate the intake of a food containing ellagitannins, but are not specific for any food. Similarly, procyanidins, metabolized by the gut microbiota produce a wide range of valerolactones and valeric acids, which have been proposed as biomarkers of procyanidin-rich foods—tea, cocoa, apple, walnuts, or wine. Considering those examples, the usefulness of these polyphenol metabolites as single biomarkers for dietary intake appears limited. For this reason, a multi-metabolite biomarker panel (MBP) was recently proposed as a tool to capture dietary exposure and improve the accuracy and precision of dietary assessment [62]. This concept has been successfully applied in a number of studies relating the urine metabolite profile to the intake of walnuts [59], cocoa [60], and bread [61]. The results from the aforementioned studies showed that MBP performs better than unique compounds in terms of predicting dietary exposure. The present review thus considered single metabolites acting as BFI, as well as the possibility of using multiple metabolite panels for assessing the intake of berries and grape.

This review has been performed in the frame of the FoodBAll project (Food Biomarkers Alliance, http://www.foodmetabolome.org/) funded by the Joint Programming Initiative “A Healthy Diet for a Healthy Life” (JPI-HDHL).

## Methods

### Selection of food groups

For this review, grapes (*Vitis vinifera*) together with raisins (i.e., dried grapes) were selected as widely consumed members of the *Vitaceae* family. The second family of fruits included in this work was botanical berries: blueberries, cranberries, blackcurrant and redcurrant, and commonly called berries: strawberries and *Rubus* fruits (raspberries and blackberries). The selection of berry types was based on the frequency of consumption reported in the FoodEx database [42] and in the literature [110]. The chokeberries and goji berries, as consumed less frequently, were not included in this review.

### Search for relevant FIB research papers

**Fig. 1:**
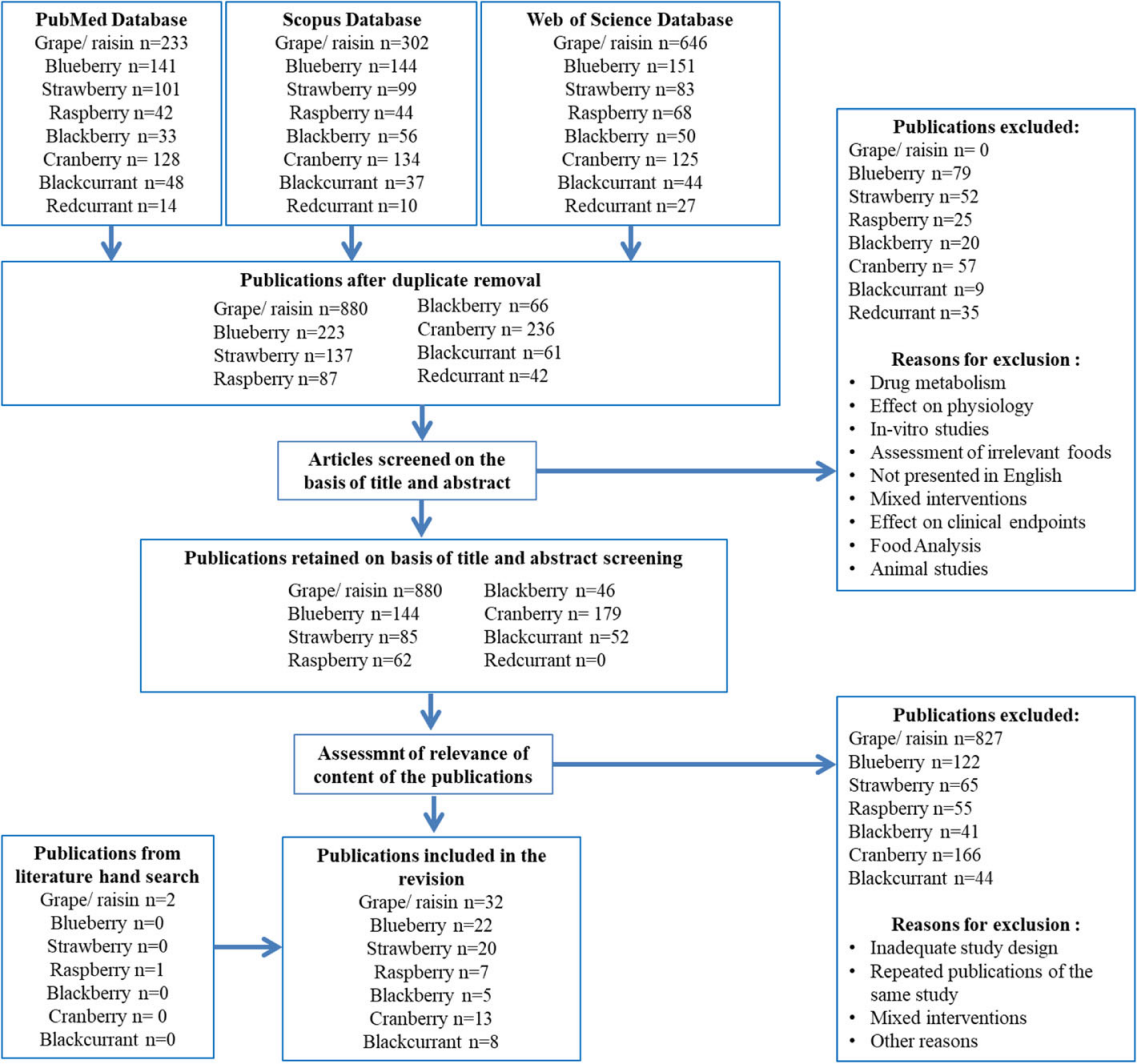
Flow diagram of study selection according to the BFIRev procedure

An extensive literature search was carried out to collect all available information on the existing and new candidate BFIs for the selected fruits. The FIBRev protocol (Food Intake Biomarker Reviews) elaborated with guidance of the PRISMA statement (Preferred Reporting Items for Systematic Reviews and Meta-Analyses) and described in Pratico et al. [128] was followed. Briefly, a primary search was performed in the three databases Scopus, PubMed central, and Web of Science to identify compounds that are already used as or may represent potential BFIs. Search for each berry type was performed independently. The name of the specific fruit and its botanical genus, i.e (strawberr* OR *fragaria*), (blackberr* OR *rubus*), (raspberr* OR *rubus*), (redcurrant OR *ribes*), (blackcurrant OR *ribes*), (blueberr* OR *vaccinium*), (cranberr* OR v*accinium*), and (grape* OR rasin*) along with the common keywords: AND (urine or plasma or serum or excretion or blood) AND (human* OR men OR women OR patient* OR volunteer* OR participant*) AND (biomarker* OR marker* OR metabolite* OR biokinetics OR biotransformation OR pharmacokinetics OR bioavailability OR ADME) AND (Intake OR meal OR diet OR ingestion OR administration OR consumption OR eating OR drink*) were applied. Keywords were used in the fields [Topic], [All fields], and [Article Title/Abstract/Keywords] for Web of Science, PubMed, and Scopus, respectively. All searches were carried out in March 2016 and updated in December 2019. Only papers in English language were considered eligible, and no restriction on the date of publication was applied. Articles showing results of human intervention studies (randomized controlled trials, acute, short-term or long-term studies) or observational studies (cohort, case-control, cross-sectional studies) were considered eligible. A first selection of papers was performed according to abstract and title relevance. Full texts were obtained for the selected articles and further assessed for eligibility according to their relevance in determining BFIs for grapes and berry fruits. Some of the publications found in the reference list of the selected articles were also included at this stage. The main exclusion criteria were papers that discussed the health effects and in-vitro studies. The research papers identifying or using potential biomarkers of intake were selected according to the process outlined in Fig. 1.

### Identification and characterization of candidate BFIs

A secondary search allowed to retrieve relevant information to assess the quality of every individual candidate biomarker, regarding specificity, biokinetics, dose–response relationship, robustness, and reliability of the method of analysis, in order to validate its use as BFI according to the scoring validating scheme established by Dragsted et al. [40].

The name of the candidate biomarkers and their synonyms were queried in the previously mentioned databases along with AND (biomarker* OR marker* OR metabolite* OR biokinetics OR biotransformation OR pharmacokinetics OR bioavailability OR ADME). Additionally, the compounds were searched manually in the online databases HMDB (https://www.hmdb.ca), FooDB (http://foodb.ca/), Phenol-Explorer (http://phenol-explorer.eu/), Dictionary of Food Compounds (http://dfc.chemnetbase.com/faces/chemical/ChemicalSearch.xhtml), Duke’s phytochemical and ethnobotanical databases (https://phytochem.nal.usda.gov/phytochem/search), eBASIS (http://ebasis.eurofir.org/Default.asp), Knapsack (http://kanaya.naist.jp/knapsack_jsp/top.html), and PhytoHub (http://phytohub.eu) to determine all the possible dietary or metabolic origins of the candidate BFIs.

Specific and non-specific metabolites were discussed in the text, while only the most plausible candidate BFIs have been reported in Table 1 including the information related to study designs and analytical methods. The non-retained compounds are listed in Table 2, with main reasons for exclusion and references for an exhaustive presentation of the results. The tables have been reviewed and agreed upon by all authors.

**Table 1:** List of studies reporting candidate biomarkers for grape/raisin and berry consumption

Dietary factor	Study design	Number of subjects	Analytical method	Sample type	Discriminating metabolites/candidate biomarkers	Reference(s)
Grapes and raisins						
Red grapes (50–200 g)	Single-dose study, short-term controlled study (4 days), crossover design	6 (3 F, 3 M)	NMR	Urine (0–24 h after intake)	• Tartaric acid	[66]
		19 (9 F, 10 M)	NMR	Urine (fasting & 24–h urine)		
Grape juice (200–1200 mL)	Single dose study	1 M	LC-MS	Urine (0–4 h after intake)	• Resveratrol	[107]
Grape juice (12–18 oz)	Intervention study	2 M	NMR	Urine (overnight)	• Tartaric acid	[96]
Grape juice (28 mL)	Intervention study	23 (not specified)	LC-MS	Urine (overnight)	• Tartaric acid	[97]
Red grape juice (1 L)	Single-dose study, crossover design	11 M	GC-MS	Plasma (0–24 h after intake) Urine (0–24 h after intake)	• *cis*-Resveratrol • *trans*-Resveratrol	[121]
Red grape juice (Concord) (350 mL)	Single-dose study	12 (7 F, 5 M)	GC-MS	Urine (0–24 h after intake)	• Tartaric acid	[158]
Grape pomace beverage (500 mL)	Crossover, placebo-controlled	12 (6 F, 6 M)	LC-MS	Urine	• *cis*-Resveratrol-3-*O*-glucuronide, • *cis*-Resveratrol-3-*O*-sulfate, • *cis*-Resveratrol-4′′-*O*-glucuronide, • *cis*-Resveratrol- 4-*O*-sulfate • *trans*-Resveratrol-3-*O*-glucuronide, • *trans*-Resveratrol-3-*O*-sulfate, • *trans*-Resveratrol-4′′-*O*-glucuronide, • *trans*-Resveratrol-4-*O*-sulfate • Dihydroresveratrol-3-*O*-sulfate, • Dihydroresveratrol-4-*O*-sulfate, • Dihydroresveratrol-sulfoglucuronide	[150]
Grape skin extract beverage (187 mL)	Single dose & long-term study (2 weeks), crossover, placebo-controlled	26 (13 F, 13 M)	UPLC MS/MS	Urine: 0–4 h after intake (acute study) and 24-h urine (long-term study)	• *trans*-Resveratrol-3-*O*-glucuronide, • *trans*-Resveratrol-4′-*O*-glucuronide, • *trans*-Resveratrol-3′-*O*-sulfate, • *trans*-Resveratrol-4′-*O*-sulfate, • *trans*-Resveratrol-3,4′-*O*-disulfate • *cis*-Resveratrol-3-*O*-glucuronide, • *cis*-Resveratrol-4′-*O*-glucuronide, • *cis*-Resveratrol-3′-*O*-sulfate, • *cis*-Resveratrol-4′-*O*-sulfate • Resveratrol sulfoglucuronide • *trans* and *cis*-piceid • Piceid glucuronide, • Piceid sulfate • Dihydroresveratrol-3-*O*-sulfate, • Dihydroresveratrol-4-*O*-sulfate, • Dihydroresveratrol-sulfoglucuronide	[143]
Grape extract tablets (15 tablets with 400 mL of water)	Single-dose study, parallel design	3 M	LC–ESI-MS/MS	Plasma (0–48 h after intake) Urine (0–48 h after intake)	• *trans*-Resveratrol-3-*O*-glucuronide (u, p), • *trans*-Resveratrol-4′-*O*-glucuronide (u, p), • *trans*-Resveratrol-3-*O*-sulfate (u), • *trans*-Resveratrol-4′-*O*-sulfate (u) • *cis*-Resveratrol-3-*O*-glucuronide (u, p), • *cis*-Resveratrol-4′-*O*-glucuronide (u, p) , • *cis*-Resveratrol-3-*O*-sulfate (u), • *cis*-Resveratrol-4′-*O*-sulfate (u) • *trans*- and *cis*-piceid (u) • piceid-glucuronide (u, p), • piceid sulfate (u) • Dihydroresveratrol-glucuronide (u, p), • Dihydroresveratrol-sulfate (u)	[142]
Resveratrol (250 mg), grape seed extract (900 mg) vs grape juice (20 oz)	Pharmacokinetic challenge, 10 days with repeated doses	7 M, 5 F	LC-MSMS	Blood: 0.5 h, 1 h, 1.5 h, 2 h, 2.5 h, 3 h, 4 h, 5 h, 6 h	• Resveratrol	[117]
Blueberry						
Bluberry extract 25 g, single-dose kinetic study	Single-dose study	10	LC-ESI-MS- Orbitrap Untargeted study	Serum: 30, 60, 120, 240, and 360 min Urine: 30, 60, 120, 240 and 360 min	• Delphinidin hexoside (u)* • Cyanidin hexoside (u)*	[2]
300 g of blanched vs unblanched blueberries	Randomized crossover design	10 M	HPLC-UV	Blood plasma: 0 h, 1 h. 2 h, 24 h	• Malvidin-3-glucoside (p) • Cyanidin-3-glucoside (p) • Phenyl-γ-valerolactone metabolite	[37]
Blueberry 250 mL juice	Single-dose kinetic study	17 (13 F, 4 M)	HPLC-ESI-MS/MS	Urine: 0 h, every void during 24 h	• Cyanidin metabolites (including glucuronide and methyl glucuronide) • Pelargonidin metabolites • Delphinidin (including glucuronide and methyl glucuronide) • Malvidin (including glucuronide and methyl glucuronide) • Peonidine (including glucuronide and methyl glucuronide) • Petunidin (including glucuronide and methyl glucuronide)	[81]
250 mL of blueberry juice per day, for 28 days (216 mg (448 μmol) cyn 3-glucoside)	Randomized one-arm study	17 (13 F, 4 M)	HPLC-ESI-MS/MS	Urine 24 h: days 0, 7th, 14th, 28th, and 36th	• Cyanidin glucuronide • Delphinidin glucuronide • Malvidin arabinose • Malvidin glucuronide • Malvidin glycoside • Malvidin methyl glucuronide • Pelargonidin glucuronide • Pelargonidin methyl glucuronide • Pelargonidin methyl glycoside • Peonidin glucuronide • Peonidin glycoside • Peonidin methyl glucuronide • Petunidin • Petunidin glucuronide • Petunidin methyl glucuronide	[83]
Blueberry powder in combination with placebo oil and fish oil. Powder dose corresponded to 25g fresh fruit	Randomized, double blind, parallel groups, placebo-controlled trial	76	HPLC-MS	Urine, morning samples, after overnight fasting, day 0, day 12th	• Total antocyanins including glucuronide conjugates	[106]
blueberry 100g (containing 1.2 g antocyanins)	Single-blind crossover with control meal	5 (not specified)	HPLC-UV	Blood serum: 0 h, 1 h, 2 h, 3 h, 4 h	• Cyanidin-3-glycosides • Delphinidin-3-glycosides • Malvidin-3-glycosides • Petunidin-3-glycosides	[103]
300 mL blueberry extract	Single-dose crossover	5 M	HPLC-UV	Urine: 0 h, 60 min, 120 min, 180 min, 240 min, 300 min	• Cyanidin-3-glycosides • Delphinidin-3-glycosides • Malvidin-3-glycosides • Petunidin-3-glycosides	[105]
10 g bilberry extract	Single dose	10 F	HPLC-MS/MS	Urine: 0–2 h, 2–4 h, 4–8 h, Blood: 0–2 h, 2–4 h, 4–8 h, 8–24 h Ileostomy fluid: 0**–**1 h, 1–2 h, 2–4 h, 4–6 h, 6–8 h	• Malvidin glucuronide • Peonidin glucuronide	[112]
189g frozen blueberry blended with 315ml water	Parallel randomized study	5 F	HPLC-DAD	Blood plasma 10 min, 20 min, 30min, 45 min, 1 h, 2 h, 4 h, 6 h, and 24 h postprandial Urine: 0 h, 0–2 h, 2–4 h, 4–6 h, 6–8 h, 8–12 h, 12–24 h	• Cyanidin-3-glycosides • Cyanidin-3-glucoside monoglucuronide • Delphinidin-3-glycosides • Malvidin-3-glycosides • Petunidin-3-glycosides	[178]
25 g freeze dried wild blueberry powder	Single blind, randomized, two-arm crossover-controlled study	6 M, 6 F	UHPLC-Q-TOF-MS^2^; UHPLC-QQQ-MS^2^	Blood: 0.25 h, 0.5 h, 1 h, 2 h, 4 h, 6 h, 8 h, 10 h, 24 h fasting	• Malvidin-3-(6′′-acetyl-glucoside) • Chlorogenic acid • Cyanidin 3-arabinose • Cyanidin-3-galactoside • Cyanidin-3-glucoside • Cyanidin glucuronide • Delphinidin-3-arabinose • Delphinidin-3-galactoside • Delphinidin-3-glucoside • Delphinidin glucuronide • Malvidin-3-arabinose • Malvidin-3-galactoside • Malvidin-3-glucoside • Peonidin-3-arabinose • Peonidin-3-galactoside • Peonidin-3-glucoside • Peonidin glucuronide • Petunidin-3-arabinose • Petunidin-3-galactoside • Petunidin-3-glucoside • Petunidin glucuronide	[185]
Strawberry						
300 g Fresh strawberries (fs) and stored strawberries (ss)	Single-dose,crossover	13 (not specified)	LC-MS/MS	Blood plasma: 0.5, 1, 2, 3, 5, and 8 h Urine: 0–2, 2–4, 4–6, 6–8, 8–12, and 12–24 h	• Pelargonidin (u) • Pelargonidin glucoside (u) • Pelargonidin glucuronide (u)	[4];
Strawberry powder: 10 g, 20 g, 40 g	Single-center, randomized, single-blind, four-arm, crossover	5 (not specified)	Q-TOF LC/MS and LC-MS/MS	Blood plasma: 0 h, 30 min, 60 min, 90 min, 120 min, 180 min, 240min, 300 min, 360 min	• Pelargonidin-*O*-glucuronide • Pelargonidin sulfate • Pelargonidin glycosides • Cyanidin glycosides	[5]
50 g Daily of freeze-dried strawberry powder for 4 weeks	4-week one-arm study	16 F	HPLC-UV	Fasting serum and plasma	• Ellagic acid	[9]
500 g Strawberries: integrated food production vs organic production	Open-label crossover design with run-in and washout periods and consisted of 8 visits at the 0th, 10th, 25th, 40th, 50th, 65th, 80th, and 90th day of observation	33 (16 M; 17 F)	HPLC-UV/VIS, HPLC-ECD	Fasting plasma and spot morning urine	• Urolithin A	[14]
Strawberry juice, 55 g of fruits	Single-intake study	3 M, 3 F	HPLC-MS	Urine: 2 h, 4 h, 6 h, 8 h, and 24 h	• Pelargonidin-3-glucoside • Pelargonidin-3-rutinoside • Pelargonidin glucuronide	[20]
Strawberry puree 100 g, 200 g, 400 g	Dose–response crossover study dosing	12 (not specified)	HPLC-UV-MS	Urine: 0, 2, 4 ,6, 8, 10, 12, and 24 h	• Pelargonidin-3-glucoside • Pelargonidin glucuronide (3 isomers)	[22]
250 g Strawberry	Randomized, 4-arm, not crossover.	40 (20 M, 20 F)	LC-MS/MS	Urine: 8, 16, 32, 40, and 56 h	• Urolithin B • Urolithin B glucuronide	[25, 26]
Strawberries (150g)	Randomized, controlled, single-blinded, three-way crossover	16 M (overweight, healthy)	LC-ESI- Q-TOF-MS	Urine: 0 h, 0–1 h, 1–2 h, 2–24 h	• Non-specific metabolites of flavonols • Furaneol glucuronide* • Furaneol sulfate* • Mesifurane sulfate*	[31]
Strawberry 10 g dry powder	Single-centre, randomized, single-blind, placebo controlled, crossover	24 overweight adults (14 F, 10 M)	LC-MS/MS	Blood plasma: 0, 30, 60, 90, 120, 180, 240, 300, 360 min	• Pelargodnidin sulfate • Pelargonidin-3-glucoside • Pelargonidin-3-glucuronide	[41]
200 g strawberries (179 mol pelargonidin-3-glucoside)	Single dose, dose–response study	6 (3 F, 3 M)	HPLC-ESI-MS-MS, and HPLC-UV	Urine: 0–2, 2–4, 4–6, 6–8, 8–12, and 12–24 h	• Pelagonidin monglucuronide (3 isomers) • Pelargonidin • Pelargonidin monosulfate • Pelargonidin-3-glucoside	[48]
Frozen strawberries (250 g)	3 weeks intervention, one arm	21 F	LC-MS/MS	Blood plasma and urine (morning urine, 0–12 h, 12–24 h, washout urine)	• 2,5-dimethyl-4-hydroxy-3-[^2^H]furanone glucuronide (furaneol glucuronide) (p, u) • Pelargonidin-glucuronide (3 isomers) (p, u) • Pelargonidin-3-glucoside (p) • Urolithin A glucuronide (p, u)	[74];
100 g, 200 g, 300 g, or 400 g portion of fresh strawberries	Randomized four phase crossover design, dose-response	10 (3M, 7F)	HPLC-UV-MS	Urine: 0–2, 2–4, 4–6, 6–8, and 8–24 h	• Pelagonidin monglucuronide (3 isomers) • Pelargonidin • Pelargonidin monosulfate • Pelargonidin-3-glucoside	[75, 76];
200 g of strawberries, containing 222 μmol of pelargonidin-3-*O*-glucoside	Single-dose, crossover study. Treatment vs control	8 (6 M, 2 F)	HPLC-PDA-MS2	Blood plasma (0, 0.5, 1, 2, 3, 4, 6, 8, and 24 h) and urine (0–2, 2–5, 5–8, and 8–24 h)	• Pelargonidin-glucuronide (4 isomers) (p, u) • Pelargonidin-3-*O*-glucoside (p, u) • Pelargonidin-O-monosulfate (u) • Pelargonidin (u)	[113]
Dry strawberry powder: 10 g, 20 g, 40 g, and placebo 0 g	A randomized, single-center, single-blinded, four-arm, placebo-controlled, 6-h postprandial, crossover study	25 (not specified)	HPLC-Triple Quandrupole MS	Blood plasma 0, 30, 60, 90, 120, 180, 240, 300, and 360 min,	• Pelargonidin-3-*O*-glucoside • Pelargonidin glucuronide • Cyanidin-3-*O*-glucoside	[126]
500 g Daily strawberry consumption for 9 days	Open label, treatment vs control with a run-in and wash-out period, consisted of 7 visits	14 (not specified)	HPLC-UV/VIS, HPLC-ECD	Fasting plasma and spot morning urine	• Urolithin A	[129]
2.5 kg Spanish strawberry and 3.0 kg Italian strawberry	Two groups, parallel, not crossover	6 (4 M, 2 F)	LC-ESI-MS/MS	Urine: 24 h	• 2,5 dimethyl-4-hydroxy-3[^2^H]furanone • 2,5 dimethyl-4-hydroxy-3[^2^H]furanone glucuronide	[141]
Strawberry drink: 132 g of fresh strawberries (34.7 ± 0.5 mg anthocyanins per drink)	Within-subjects crossover design, delivering one strawberry drink and two control drinks	24 (not specified)	UPLC-Triple Quandrupole MS	Blood plasma, from 0 to 10 h after meal	• Pelargonidin-3-*O*-glucoside • Pelargonidin glucuronide • Pelargonidin-3*-O*-rutinoside • Cyanidin-3-*O*-glucoside • Cyanidin-3-*O*-rutinoside	[147]
12 g of freeze-dried strawberry powder	Double-blinded, 2-arm, placebo-controlled 90-day feeding trial	38	LC-MS/MS	Blood: Day 1 fasting blood; Day 45: fasting blood and 2 h postprandial Day 90: fasting blood and 2 h postprandial	• Urolithin A glucuronide • Isourolithin A glucuronide • Urolithin B glucuronide • Pelargonidin-3-glucoside • Pelargonidin-3-rutinoside • Pelargonidin glucuronide	[148]
200 g of fresh strawberries or 200 g strawberry puree	4-week randomized study	20 (8 M, 12 F)	LC-MS/MS	Urine: 0−8, 8−32, 32−56, 56−80, and 80−92 h	• Urolithin A glucuronide • Urolithin A • Urolithin B • Urolithin B glucuronide	[165]
Strawberry milk beverage and strawberry water beverage; 12 g of powder in each drink	Single-center, randomized, single-blinded, crossover study	9		Blood 0 h, 0.25 h, 0.5 h, 1 h, 1.5 h, 2 h, 3 h, 4 h, 5 h, 6 h	• Pelargonidin-3-glucoside • Pelargonidin-3-rutinoside • Pelargonidin glucuronide	[179]
Raspberry						
300 g of raspberries	Acute, 1-arm study	10 healthy persons (6 M, 4 F); 4 with ileostomy	HPLC-PDA-MS2	Blood plasma: 0, 1.0, 1.5, 2.0, 3.0, 5.0, 6.5, 7.5, and 24 h Urine: 0–4 h, 4–7 h, 7–24 h, 24–32 h, and 32–48 h Ileal fluid: 0–4 h, 4–7 h, 7–24 h, and 24–48 h	• Cyanidin-3-*O*-(2′′-O-glucosyl)rutinoside (if, u) • Cyanidin-3-*O*-(2′′-O-xylosyl)rutinoside (if) • Cyanidin-3-*O*-glucoside (if, u) • Cyanidin-3-*O*-rutinoside (if) • Cyanidin-3-*O*-sophoroside (if, u) • Ellagic acid(if, u) • Ellagic acid-*O*-glucuronide (u) • Pelargonidin-3-*O*-(2′′-*O*-glucosyl)rutinoside (if) • Pelargonidin-3-*O*-glucoside (if) • Pelargonidin-3-*O*-sophoroside (if) • Sanguiin H-6 (if) • Urolithin A glucuronide (u)	[70]
200 g, 400 g of raspberries, + placebo	3-arm double-blind randomized controlled crossover	10 M	UPLC-QTOF	Blood: 0 h, 2 h and 24 h postprandial	• Urolithin A • Urolithin A-3-glucuronide • Urolithin A-sulfate • Urolithin B glucuronide • (Iso)urolithin A • (Iso)urolithin A-3-glucuronide	[78]
300 g of homogenized raspberries	Acute, single dose	9 (4 M, 5 F)	UHPLC-MSn and UHPLC-MS2	Urine: 0–4 h, 4–8 h, 8–24 h, 24–32 h, 32–48 h Blood plasma: 0.5 h, 1 h, 1.5 h, 2 h, 3 h, 4 h, 5 h, 6 h, 8 h, 24 h	• (Iso)urolithin A-sulfate-*O*-glucuronide (u, p) • Isourolithin A-*O*-glucuronide (u, p) • Peonidin-3-*O*-glucoside (u) • Urolithin A (u) • Urolithin A-*O*-glucuronide (u, p) • Urolithin A-sulfate (u, p) • Urolithin B (u) • Urolithin B-3-*O*-glucuronide (u, p) • Dimethylellagic acid-O-glucuronide (u, p) • Cyanidin-3*-O*-glucoside (u, p) • Cyanidin-3-*O*-glucuronide (p) • Peonidin-3-*O*-glucoside (u)	[98]
Intragastric dosage of raspberry ketone, 1 mmol/kg	Single dose	(not specified)	GC-MS after derivatization	Urine of rat,rabbit, and guinea pig	• 2-(4-hydroxyphenyl)ethanol • 4-(4-hydroxyphenpl)butan-2-one • 4-hydroxybenzoic acid • 4-hydroxyphenylacetic acid • 4-(4-hydroxyphenyl)butan-2-ol • 4-(4-hydroxy-3-methoxyphenyl)butan-2-one • 4-hydroxy-4-(4-hydroxyphenyl)butan-2-one • 3-(4-hydroxyphenyl)propionic acid • 4-(3,4-dihydroxyphenyl)butan-2-one • 4-(4-hydroxy-3-methoxpphenyl)butan-2-ol • 4-(4-hydroxyphenyl)butan-2,3-diol • 4-(3,4-dihydroxyphenyl)butan-2-ol • 1-hydroxy-4-(4-hydroxphenyl)butan-2-one • 4-(4-hydroxyphenyl)butan-l,2-diol	[156]
125 g raspberries per day for 4 weeks.	Not specified	3	UHPLC-QTOF (MS/MS); UHPLC-QQQ (MS/MS)	Blood: 0 h, 0.5 h, 1 h, 2 h, 3 h, 4 h, 5 h, 5.5 h, 6 h, 7 h, 8 h, and 24 h Urine: − 12 h–0 h; 0–4 h, 4–8 h, 8–24 h, 24–36 h, 36–48 h	• Cyanidin-3-*O*-sophoroside • Cyanidin-3-*O*-(2G glucosylrutinoside) • Cyanidin-3-*O*-glucoside • Pelargonidin-3-*O*-sophoroside • Cyanidin-3-*O*-rutinoside • Cyanidin-3-*O*-glucuronide • Methyl cyanidin-3-*O*-sophoroside • Peonidin-3-*O*-glucoside • Urolithin A glucuronide • Urolithin B glucuronide • Urolithin A	[184]
Blackberry						
200 g of blackberries (containing 960 μmol of anthocyanins expressed as cyanidin 3-glucoside equivalents)	Acute, single dose, kinetic study	5 (3 F, 2 M)	HPLC-ESI-MS-MS and HPLC with UV-vis	Urine: 0–2, 2–4, 4–6, 6–8, 8–12, and 12–24 h	• Cyanidin glycosides • Cyanidin glucuronide • Cyanidin diglucuronide • Cyanidin sulfate • Peonidin glycosides • Peonidin glucuronide, • Peonidin-3-glucoside glucuronide	[47]
250 mL of tropical highland blackberry juice	Acute study, repeated over the year, no placebo, different kinetics design	26 (16 F, 10 M)	UPLC-DAD/ESI-Q-TOF/MS2	Spot urine samples	• Urolithin A • Urolithin B • Urolithin C • Urolithin D • Urolithin M5 • Methyl urolithin A • Urolithin glucuronide A • Urolithin glucuronide B • Urolithin glucuronide C • Urolithin glucuronide D • Ellagic acid • Ellagic acid dimethyl ether glucuronide • Ellagic acid dimethyl ether	[64, 65]
Freeze-dried blackberries: 32 g for females and 45 g for males	26 weeks pilot study with daily consumption of supplement	20	LC-MS/MS	3 h pooled urine collected in the morning: week 0, week 12, week 26	• Urolithin A glucuronide • Urolithin A-sulfate • Dimethylellagic acid glucuronide	[89]
250 g of blackberries	Randomized and crossover intervention	18 (not specified)	HPLC-DAD	Blood plasma: 15, 30, 60, and 12 min Urine: 0, 120 min	• Cyanidin glycosides (u) • Cyanidin glucoside glucuronide (u, p) • Cyanidin glucoside sulfate (p, u) • Cyanidin glucuronide (two isomers) (p, u) • Methyl cyanidin glucoside glucuronide (p, u) • Methyl cyanidin glucuronide (p, u)	[100]
20 g freeze-dried blackberry powder, three times a day, for 9 weeks	One-arm study, patients with colorectal cancer	20 (17 M, 3 F)	UHPLC-MS/MS, Orbitrap LTQ, and GC-MS	Blood plasma, urine at baseline and after treatment	• Cyanidin glycosides (u)	[123]
45 g of freeze-dried blackberries	One arm, 1 week study with 45 g of meal daily	10 M	LC-MS/UV-Vis; HPLC-ESI-MS/MS	Urine: 4 h, 8 h, 12 h, at 1st and 7th day	• Cyanidin glycosides	[161]
Cranberry						
480 mL cranberry Juice	Acute, single-dose, kinetic study	15 (13 M, 2 F)	LC-Diode array/ ion trap MS/MS	Blood plasma: 0 h, 1 h, 2 h, h, 4 h urine: 0–4 h	• Cyanidin-3-glycosides (u, p) • Malvidine-3-glycosides (u, p) • Peonidin-3-glycosides (u, p)	[108];
200 mL cranberry juice	Acute, single-dose, kinetic study	11 (9 M, 2 F)	HPLC-ESI-MS-MS	Urine 24 h	• Cyanidin-3-glycosides • Malvidine-3-glycosides • Peonidin-3-glycosides	[119]
250 mL cranberry juice twice a day	Crossover, 2-arm study	17 F	UHPLC-Q-Orbitrap-HRMS	Blood: fasting base line, 2h postprandial on third consumption day	• 5-(trihydroxphenyl)-γ-valerolactone* • Phenolics derivatives*	[94]
450 mL	Double-blind, placebo-controlled parallel trial	78	LC-MS/MS	Urine: 0 h, 3 h, 5 h, 8h postprandial	• Cyanidin-3-glucoside • Peonidin-3-glucoside • Cyanidin-3-galactoside • Peonidin-3-galactoside • Peonidin-3-arabinoside • Cyanidin-3-arabinoside	[27]
2 types of capsules of cranberry extract, intake for 7 days; 36 mg vs 12 mg extract per capsule	Randomized, double-blind, 2-arm repeated measure crossover design	13	LC-LTQ-Orbitrap XL	Urine: 0 h, 1 h, 2 h, 4 h, 6 h, 10 h, 12 h, 24 h, at day 0 and day 7th	• Hydroxyphenyl-valerolactone derivatives*	[7]
Blackcurrant						
100 g whole blackcurrant vs 50 g diluted blackcurrant syrup	Randomized two-phase crossover	10 (3 M, 7 F)	HPLC-ESI-MS	Blood plasma: 15 min, 30 min, 45 min and 1 h, 1.5 h, 2 h, 3 h, 4 h, 6 h, 8 h, 24 h, and 48 h Urine: 0–2 h, 2–4 h, 4–6 h, 6–8 h, 8–24 h and 24–48 h	• Cyanidin-3-*O*-rutinoside (u) • Delphinidin 3-*O*-rutinoside (u)	[75, 76]
250 mL black currant juice vs placebo drink	Randomized, cross-over, double-blind, placebo-controlled acute meal study	20 (11 F, 9 M)	GC-MS	urine: collected every 2 h and at 24 h	• Cyanidin-3-*O*-rutinoside • Delphinidin 3-*O*-glucoside • Delphinidin 3-*O*-rutinoside	[80]
300 mL blackcurrant extract	Acute crossover	5 M	HPLC-UV	Urine: 0 h, 60 min, 120 min, 180 min, 240 min, 300 min	• Cyanidin-3-*O*-rutinoside • Cyanidin-3-*O*-glucoside • Delphinidin 3-*O*-glucoside • Delphinidin 3-*O*-rutinoside	[105]
253 mg of blackcurrant extracts	Acute single dose	4 (3 F, 1 M)	LC-MS/MS	Blood plasma: 0 h, 0.5 h, 1 h, 2 h, 3 h, 4 h, 6 h	• Cyanidin-3-*O*-rutinoside • Cyanidin-3-*O*-rutinoside • Delphinidin 3-*O*-glucoside • Delphinidin 3-*O*-rutinoside	[114]
200 mL blackcurrant juice	Acute, single-dose, kinetic study	4 (2 M , 2 F)	HPLC-UV-VIS	Urine: 0 h, 0.5 h, 1 h, 1.5 h, 2 h, 2.5 h, 3 h, 3.5 h, 4 h, 4.5 h, 5 h	• Cyanidin-3-*O*-rutinoside • Cyanidin-3-*O*-glucoside • Delphinidin 3-*O*-glucoside • Delphinidin 3-*O*-rutinoside	[115]
1.6 g blackcurrant extract powder in 200 mL water	Acute single-dose kinetic study	5 M	HPLC-ESI-MS/MS	Plasma 0 h, 1 h, 2 h, 4 h, 6 h, 8 h Urine: 24−0 h prior to ingestion; 0−2 h, 2−4 h, 4−6 h, 6−8 h, 8−12 h, 12−24 h, 24−48 h postprandial	• Delphinidin 3-*O*-rutinoside • Delphinidin 3-*O*-glucuronide • Cyanidin-3-*O*-glucoside • Cyanidin 3-*O*-glucuronide	[139]

* Metabolites found through untargeted metabolomics experiments

*u* urine, *p* plasma, *if* illeal fluid

**Table 2:** List of non-retained compounds for grapes/raisins and berries

Food item	Compound	Biofluid	Reasons for exclusion	References
Raisin	*p*-Hydroxybenzoic acid	Plasma	Unspecific	[84]
Grape	**Anthocyanins and its derivatives:** cyanidin-3-glucoside, cyanidin-3-glucuronide, delphinidin-3-glucoside, delphinidin-3-glucuronide, malvidin-3-glucoside, malvidin-3-glucuronide, peonidin-3-glucoside, peonidin-3-glucuronide, petunidin-3-glucoside, petunidin-3-glucuronide	Plasma/ urine/ ileal fluid	Unspecific, low bioavailability	[19, 55, 15, 157]
	**Flavan-3-ol metabolites and its derivatives**: catechin, catechin glucuronide, catechin sulfate, catechin sulfate glucuronide, epicatechin, epicatechin glucuronide, epicatechin sulfate, epicatechin sulfate glucuronide, epicatechin-3-*O*-gallate, *O*-methyl-(epi)catechin-*O*-glucuronide, *O*-methyl-(epi)catechin-*O*-sulfate, gallocatechin, epigallocatechin, epigallocatechin-*O*-glucuronide, *O*-methyl-(epi)gallocatechin-*O*-sulfate, 4′′-*O*-methyl-(epi)gallocatechin-*O*-sulfate, 4-hydroxy-5-(3,4-dihydroxyphenyl)valeric acid, 4-hydroxy-5-(dihydroxyphenyl)-valeric acid glucuronide, 4-hydroxy-5-(dihydroxyphenyl)-valeric acid sulfate, 5-(3,4′-Dihydroxyphenyl)-g-valerolactone, 5-(3′,4′-dihydroxyphenyl)-ɣ-valerolactone, 5-(3′,5′-dihydroxyphenyl)-ɣ-valerolactone, 5-(dihydroxyphenyl)-γ-valerolactone glucuronide, 5-(hydroxyphenyl)-γ-valerolactone glucuronide, 5-(hydroxy-methoxy-phenyl)-γ-valerolactone glucuronide	Plasma/ urine/ ileal fluid	Unspecific, common for many fruits and vegetables	[171, 157, 158, 99, 85, 150, 117, 23, 69, 29]
	Procyanidin B1	Serum	Unspecific, common for many fruits	[149]
	**Benzoic acids and its derivatives:** gallic acid, *O*-methylgallic acid, *O*-methylgallic acid-3-*O*-sulfate, 3,4-*O*-dimethylgallic acid, hydroxybenzoic acid, hydroxy-dimethoxybenzoic acid glucuronide, 3,4-dihydroxybenzoic acid, 3-methoxy-4-hydroxybenzoic aicd, 3,5-dimethoxy-4-hydroxybenzoic aicd, syringic acid, vanillic acid, vanillic acid-glucuronide, vanillic acid 4-sulfate, isovanillic acid, homovanillic acid, homovanillic acid 4-sulfate, pyrogallol, 2-*O*-methylpyrogallol, protocatechuic-*O*-glucoside, hippuric acid, hydroxyhippuric acid	Plasma/ urine/ ileal fluid	Unspecific, common for many fruits and vegetables	[177, 79, 168, 158, 157, 131, 99, 85, 66, 150, 23, 69, 29]
	**Quercetin and its derivatives:** quercetin, quercetin glucuronide, 3′-*O*-methyl quercetin, dihydroquercetin	Plasma/ urine	Unspecific, common for many fruits and vegetables	[107, 33, 24, 150, 117]
	**Cinnamic acids and its derivtives**: caffeic acid, caffeic acid sulfate, dihydrocaffeic acid, dihydrocaffeic acid glucuronide, dihydrocaffeic acid sulfate, ferulic acid, ferulic acid sulfate, dihydroferulic acid, isoferulic acid glucuronide, isoferulic acid sulfate, dihydroferulic acid, dihydroferulic acid glucuronide, dihydroferulic acid sulfate p-coumaric acid, p-coumaric acid sulfate, m-dihydrocoumaric acid, dihydrocoumaric acid-*O*-sulfate, *trans*-caftaric acid, *trans*-coutaric acid, *trans*-fertaric acid, dihydrosinapic acid glucuronide	Plasma/ urine/ ileal fluid	Unspecific, common for many fruits and vegetables	[79, 168, 157, 150, 23, 69, 29]
	**Phenylacetic-, and phenylpropionic acids and its derivatives:** 2-hydroxyphenylacetic acid, 3-hydroxyphenylacetic acid, 4-hydroxyphenylacetic acid, 3,4-dihydroxyphenylacetic acid, 3,4-dihydroxyphenylacetic acid, hydroxyphenylacetic acid, hydroxyphenylpropionic acid, dihydroxyphenylpropionic acid, dihydrosinapic acid glucuronide, hydroxyphenylpropionic aicd, dihydroxyphenylpropionic acid	Plasma/urine	Unspecific, common for many fruits and vegetables	[177, 168, 158, 85, 150, 69]
	**Mandelic acid metabolites** mandelic acid, 4-hydroxymandelic acid, vanilmandelic acid	Urine	Unspecific	[168, 158, 69])
	**Other metabolites:** feruloylglycine, vanilloylglycine, 6-sulfatoxymelatonin, enterodiol, tyrosine	Urine	Unspecific	[79, 71, 157, 85]
	Total phenols, conjugated phenols	Plasma	Unspecific	[118, 6]
	Naringenin	Urine	Unspecific	[79]
	Urolithin A	Plasma	Unspecific, common for many fruits and nuts	[122]
Blueberry	**Anthocyanidins, and its derivatves:** cyanidin, cyanidin glucuronide, cyanidin glycoside, cyanidin methyl glucoside, cyanidin methyl glucuronide, delphinidin, delphinidin glucuronide, delphinidin glycoside, delphinidin glycoside methyl glucuronide, delphinidin methyl glucuronide, delphinidin methyl glycoside, malvidin 3-glucoside, malvidin glucuronide, malvidin glycoside, malvidin glycoside glucuronide, malvidin glycoside sulfate, malvidin methyl glucuronide, malvidin methyl glycoside, pelargonidin glycoside, pelargonidin glucuronide, pelargonidin glycoside sulfate, pelargonidin methyl glucuronide, pelargonidin methyl glycoside, pelargonidin, peonidin, peonidin glucuronide, peonidin glycoside, peonidin glycoside sulfate, peonidin methyl glucuronide, petunidin, petunidin glucuronide, petunidin glycoside, petunidin glycoside glucuronide, petunidin glycoside methyl, petunidin glycoside methyl glucuronide, petunidin methyl glucuronide	Urine, plasma	Unspecific, common for many fruits and vegetables, low bioavailability	[37, 80–83, 103, 105, 178, 112]
	**Flavan-3-ol metabolites and its derivatives**: (4r)-5-(3-hydroxyphenyl)-valerolactone-4-*O*-sulfate, hydroxyphenyl-valerolactone*, dihydroxyphenyl-valerolactone*, hydroxy-(dihydroxyphenyl) pentenoic acid glucuronide*, hydroxy-(dihydroxyphenyl) valeric acid glucuronide I *, hydroxy-(dihydroxyphenyl) valeric acid glucuronide II *, hydroxy-(dihydroxyphenyl) valeric acid sulphate*, hydroxy-(dihydroxyphenyl) valeric acid glucuronide I *, hydroxy-(dihydroxyphenyl) valeric acid glucuronide II*, hydroxy-(dihydroxyphenyl) valeric acid sulphate*	Urine, plasma	Unspecific, common for many fruits and vegetables	[49, 50, 12, 2]
	**Benzoic acids and its derivatives:** benzoic acid, 2,3-dihydroxybenzoic acid, 2,4-dihydroxybenzoic acid, 2,5-dihydroxybenzoic acid, 2-hydroxybenzoic acid, 3-hydroxybenzoic acid, 4-hydroxybenzoic acid, 3-methoxybenzoic acid-3-sulfates 4-methoxybenzoic acid-4-sulfates 3,4-dihydroxybenzaldehyde, 3,5-dihydroxybenzyl alcohol, 3,5-dimethoxybenzoic acid methyl ester, 4-methylcatechol-*O*-sulfate, gallic acid, 3-*O*-methylgallic acid, 4-methylgallic acid-3-*O*-sulfate, protocatechuic acid, catechol glucuronide*, catechol sulphate* hydroxy-(hydroxyphenyl)pentenoic acid glucuronide*, hydroxy-(hydroxy-methoxyphenyl)-pentenoic acid glucuronide I*, hydroxy-(hydroxy-methoxyphenyl)-pentenoic acid glucuronide II*, hydroxy-(hydroxyphenyl)pentenoic acid sulphate II*, hydroxy-(hydroxy-methoxyphenyl)-pentenoic acid sulphate*, hydroxy-(dihydroxyphenyl) pentenoic acid sulphate*, hydroxy-(hydroxyphenyl)pentenoic acid sulphate*, hydroxy-(dihydroxyphenyl) pentenoic acid glucuronide*, chlorogenic acid*, 1-methylpyrogallol-*O*-sulfate, 2-methylpyrogallol-*O*-sulfate, pyrogallol-*O*-1-sulfate, pyrogallol-*O*-2-sulfate 2,4,6-trihydroxybenzaldehyde, 4-hydroxybenzaldehyde, Benzoylglutamic acid hippuric acid, 2-hydroxyhippuric acid, 3-hydroxyhippuric acid, 4-hydroxyhippuric acid, α-hydroxyhippuric acid, hydroxy hippuric acid sulphate*, hydroxy-methoxy hippuric acid*	Urine, plasma	Unspecific, common for many fruits and vegetables	[37, 131–136, 2, 34, 112, 3, 12, 160, 32, 137]
	**Cinnamic acids and its derivatives and conjugates** caffeic acid, caffeic acid 3-*O*-d-glucuronide, caffeic acid 4-*O*-d-glucuronide, cinnamic acid, dihydrocaffeic acid, dihydrocaffeic acid 3-*O*-D-glucuronide, dihydrocaffeic acid 3-*O*-sulfate, dihydroferulic acid, dihydroferulic acid 4-*O*-D-glucuronide, dihydroferulic acid 4-*O*-sulfate, dihydroisoferulic acid 3-*O*-D-glucuronide, dihydroisoferulic acid 3-*O*-sulfate, ferulic acid, ferulic acid 4-*O*-glucuronide, ferulic acid 4-*O*-sulfate, hydroxy-methoxy cinnamic acid glucuronide I (ferulic acid)*, hydroxy-dimethoxy cinnamic acid glucuronide (sinapic acid)*, hydroxy-methoxy cinnamic acid glucuronide II (ferulic acid)*, trimethoxy-hydro cinnamic acid glucuronide*, trimethoxy-hydro cinnamic acid glucuronide* homovanillic acid, homovanillic acid sulfate, isoferulic acid, isoferulic acid 3-*O*-D-glucuronide, isoferulic acid 3-*O*-sulfate, isovanillic acid, isovanillic acid glucuronide, vanillic acid, vanillic acid glucuronide, vanillic acid-4-*O*-sulfate, hydroxy-methoxy benzoic acid glucuronide I (Vanillic acid)*, hydroxy-methoxy benzoic acid glucuronide II (Vanillic acid)*, hydroxy-methoxy benzoic acid hexoside (vanillic acid glucoside)*, *m*-coumaric acid, *o*-coumaric acid, *p*-coumaric acid, dihydro-*m*-coumaric acid, sinapic acid, syringic acid, hydroxy-dimethoxy benzoic acid sulphate (syringic acid)*, hydroxy-dimethoxy benzoic acid glucuronide I (syringic acid)*, hydroxy-dimethoxy benzoic acid glucuronide II (syringic acid)*	Urine, plasma	Unspecific, common for many fruits and vegetables	[49, 50, 135, 136, 132, 2, 112, 3, 185, 160, 91, 32, 137]
	**Phenyl acetic-, phenyl propionic acids, and its derivatives:** phenylacetic acid, 3,4-dihydroxyphenylacetic acid, 3-hydroxy-phenylacetic acid, 4-hydroxyphenylacetic acid, *p*-hydroxyphenyl-acetic 4-hydroxy-3-methoxyphenylpropionic acid, 3-(3′-hydroxyphenyl)-3-hydropropionic, 2-(4-hydroxyphenoxy)propionic acid, dihydroxyphenyl propionic acid glucuronide, hydroxyphenyl propionic acid sulphate	Urine, plasma	Unspecific, common for many fruits and vegetables	[49, 50, 135, 136, 134, 153, 32, 137]
	**Flavonols, its derivatives and conjugates** kaempferol, kaempferol-3-*O*-d-glucuronide, quercetin, quercetin-3-*O*-d-glucuronide Methyl-dihydro myricetin*	Urine, plasma	Unspecific, common for many fruits and vegetables	[49, 50, 135, 136, 134, 137, 2]
	**Flavan-3-ol metabolites** Catechin, epicatechin, (epi)catechin glucuronide, (epi)catechin sulfate, (epi)catechin glucuronide sulfate, (epi)catechin methyl glucuronide, (epi)catechin methyl-sulfate	Urine, plasma		[12]
	Abscisic acid*, hydroxy-abscisic acid glucuronide*, hydroxy-abscisic acid glucuronide*, hydroxy-abscisic acid*	Urine, serum		[91, 2]
Strawberry	**Ellagic acids/ urolithins, its derivatives and conjugates** ellagic acid, urolithin A, urolithin A glucuronide, urolithin B, urolithin B glucuronide	Urine, plasma	Unspecific, common for many fruits and vegetables	[9, 14, 25, 26, 74, 129, 165, 148]
	**Anthocyanidins, its derivatves and conjugates** pelargonidin, pelargonidin-3-*O*-glucoside, pelargonidin-3-*O*-rutinoside, pelargonidin glucuronide four isomers, pelargodnidin sulfate, 5-carboxypyranopelargonidin-3-*o*-beta-glucopyranoside, cyanidin-3,5-diglucoside, cyanidin-3-*O*-glucoside, cyanidin-3-*O*-rutinoside	Urine, plasma	Unspecific, common for many fruits and vegetables, low bioavailability	[4, 5, 22, 41, 48, 73–76, 113, 126, 147, 179, 20]
	**Benzoic acids, its derivatives and conjugates** 4-hydroxybenzoic acid, 3-hydroxybenzoic acid, 3,4-dihydroxybenzoic acid, gallic acid, syringic acid, 4-hydroxyhippuric acid, salicylic acid, syringic acid, gentisic acid, vanillic acid, 3,4-dihydroxybenzaldehyde, 4-hydroxybenzaldehyde,	Urine, plasma	Unspecific, common for many fruits and vegetables	[4, 5, 129, 145, 148]
	**Cinnamic acids, its derivatives and conjugates** coumaric acid, caffeic acid, homovanillic acid, ferulic acid, synapic acid, vanillic acid, *trans*-cinnamic acid, isovanillic acid	Urine, plasma	Unspecific, common for many fruits and vegetables	[4, 5, 14, 145, 148]
	**Flavonols, its derivatives and conjugates** fisetin, fisetin-3-rutinoside, kaempferol, kaempferol-3-(6′′malonylglucoside), kaempferol-3-coumaroylglucoside, kaempferol-3-glucoside, kaempferol-3-*O*-sulfate, kaempferol-glucuronide, dihydrokaempferol glucuronide, kaempferol-3-coumaroyl-glucoside quercetin, quercetin-3-(6′′-caffeoylgalactoside), quercetin-3-galactoside, quercetin-3-glucoside, quercetin-glucuronide, quercetin-rutinoside, quercetin-sulfate, methylquercetin,	Urine, plasma	Unspecific, common for many fruits and vegetables	[5, 31, 41],
	catechin	Plasma	Unspecific, common for many fruits and vegetables	[5]
	hippuric acid	Plasma	Unspecific, common for many fruits and vegetables	[148]
Raspberry	**Anthocyanidins, its derivatves and conjugates** cyanidin-3-*O*-(2′′-*O*-glucosyl)rutinoside, cyanidin-3-*O*-(2′′-*O*-xylosyl)rutinoside, cyanidin-3-*O*-glucoside, cyanidin-3-*O*-rutinoside, cyanidin-3-*O*-sophoroside, cyanidin-3-*O*-glucuronide, pelargonidin-3-*O*-(2′′-*O*-glucosyl) rutinoside, pelargonidin-3-*O*-glucoside, pelargonidin-3-*O*-sophoroside, peonidin-3-*O*-glucoside	Urine, plasma ileal fluid	Unspecific, common for many fruits and vegetables, low bioavailability	[70, 98, 184]
	**Ellagic acids/ urolithins, its derivatives and conjugates** ellagic acid, ellagic acid-*O*-glucuronide, dimethylellagic acid-O-glucuronide, urolithin A, urolithin A-*O*-glucuronide, urolithin A-sulfate, isourolithin A-*O*-glucuronide, urolithin B, urolithin B-3-*O*-glucuronide, (iso)urolithin A-sulfate-O-glucuronide	Urine, plasma	Unspecific,, common for many fruits and nuts	[70, 98, 184]
	sanguiin H6	Ileal fluid	low bioavailability, low concentrations in biological fluids	[70]
	**Cinnamic acids and its derivatives:** caffeic acid, caffeic acid sulfate, caffeic acid-3′-sulfate, caffeoyl sulfate*, ferulic acid, ferulic acid-4′-sulfate, ferulic acid-4′-*O*-glucuronide, isoferulic acid-3’-*O*-glucuronide, isoferulic acid-3’-sulfate, isoferulic acid-3′-*O*-glucuronide, dihydrocaffeic acid-3′-sulfate, dihydroferulic acid glucuronide, dihydroferulic acid sulfate	Urine, plasma	Unspecific, common for many fruits and vegetables	[95, 98, 184]
	**Benzoic acids and its derivatives:** 3-hydroxybenzoic acid-4-sulfate, 4-hydroxybenzoic acid, 4-hydroxybenzoic acid-3-sulfate, 2,6-dihydroxybenzoic acid, 2,3-dihydroxybenzoic acid, 2,4-dihydroxybenzoic acid, 3,4-dihydroxybenzoic acid, methyl-4-hydroxybenzoic acid, methyl-3,4-dihydroxybenzoic acid, 4-hydroxybenzoic acid glucuronide, 3-hydroxybenzoic acid glucuronide, gallic acid hippuric acid, 4-hydroxyhippuric acid, 3-hydroxyhippuric acid , 3,4-dihydroxybenzoic acid sulfate 3,4-dihydroxybenzaldehyde, 4-hydroxybenzaldehyde glucuronide, 4-hydroxybenzaldehyde	Urine, plasma	Unspecific, common for many fruits and vegetables	[95, 98, 184]
	**Phenyl acetic-, phenyl propionic acids, and its derivatives:** 3,4-dihydroxyphenyl acetic acid, 3′-methoxy-4′-hydroxyphenylacetic acid, 3,4-dihydroxyphenylacetic, 4-hydroxyphenylacetic acid, 3-hydroxyphenylacetic acid, 2-hydroxyphenylacetic acid, 3-hydroxyphenylpropionic acid sulfate, 3-hydroxyphenylpropionic acid	Urine, plasma	Unspecific, common for many fruits and vegetables	[98, 184]
	Vitamin C	Urine	Unspecific, common for many fruits and vegetables	[95]
	naringenin glucuronide	Urine	Unspecific, common for many fruits	[95]
	methyl-epicatechin sulfate* catechin glucuronide	Urine	Unspecific, common for many fruits and vegetables	[95, 184]
	**Endogenous metabolites**: indoxyl sulfate, adenine, alanine, asparagine, betaine, carnitine, citrate, formate, glutamine, glycine, histidine, lysine, *N*-phenylacetylglycine, phenylacetate, serine	Plasma	Endogenous metabolites	[86]
Blackberry	**Anthocyanins and its derivatives:** cyanidin 3-glucoside, cyanidin 3-xyloside, cyanidin glucuronide, cyanidin diglucuronide, cyanidin sulfate, 4′-methyl-cyanidin-3-glucoside, cyanidin 3-glucoside, cyanidin 3-rutinoside, cyanidin glucoside glucuronide, cyanidin glucoside sulfate, cyanidin glucuronide (two isomers), methyl cyanidin glucoside glucuronide, methyl cyanidin glucuronide, cyanidin-3-rutinoside, cyanidin-3-xylosylrutinoside, cyanidin-3-glucoside, cyanidin-3-sambubioside, cyanidin 3-rutinoside, cyanidin 3-sambubioside, cyanidin 3-xylosylrutinoside, cyanidin-3-O-glucoside, methyl cyanidin 3-glucoside, methyl cyanidin 3-rutinoside, methyl cyanidin 3-sambubioside, methyl cyanidin 3-xylosylrutinoside, peonidin-3-glucoside, peonidin 3-xyloside, peonidin glucuronide, peonidin-3-glucoside glucuronide, 3′methyl-cyanidin-3-glucoside	Urine, plasma	Unspecific, common for many fruits and vegetables	[47, 100, 123, 161];
	**Ellagic acids/ urolithins, and its derivatives:** ellagic acid, urolithin A, urolithin A glucuronide, urolithin B, urolithin B glucuronide, urolithin C, urolithin C glucuronide, urolithin D, urolithin D glucuronide, urolithin M5, methyl urolithin A, ellagic acid dimethyl ether glucuronide, ellagic acid dimethyl ether, hexahydroxydiphenyl	Spot urine	Unspecific, common for many fruits and nuts	[64, 65]
	**Others metabolites** (2,3-dihydroxyisovaleric acid, 2,5-furandicarboxylic acid, 2-isopropylmalate, 2-oxindole-3-acetate, 3-[3-(sulfooxy)phenyl]propanoic acid, 3-hydroxyphenylacetic acid, 3-methylhistidine, 4-acetylphenol sulfate, 5-hydroxymethyl-2-furoic acid, abscisic acid, anthranilate, chiro-inositol, cinnamoylglycine, *cis*-aconitate, cortisol, cortisone, methyl-beta-glucopyranoside, *n*-(2-furoyl)glycine, *N*-acety-3-methylhistindine, *N*-acetylproline, xylitol, xylose, glycerol 3-phosphate	Plasma	Unspecific, common for many fruits and vegetables	[123]
	**Benzoic acids, and its derivatives:** 4-methylcatechol sulfate, catechol, catechol sulfate, hippuric acid, 3-hydroxyhippuric acid, 4-hydrixyhippuric acid, 2,4,6-trihydroxybenzoic acid	Urine, plasma	Unspecific, common for many fruits and vegetables	[64, 65, 123]
Cranberry	**Anthocyanins and its derivatives:** cyanidin-3-arabinoside, cyanidin-3-galactoside, cyanidin-3-glucoside, malvidine-3-glucoside, peonidin-3-arabinoside, peonidin-3-galactoside, peonidin-3-glucoside	Urine, plasma	Unspecific, common for many fruits	[108] [119];,
	**Cinnamic acids and its derivatives:** ferulic acid, ferulic acid sulfate*, dihydro ferulic acid, dihydro ferulic acid 4-*O*-d-glucuronide, dihydro ferulic acid 4-*O*-sulfate, dihydro isoferulic acid 3-*O*-d-glucuronide, dihydro isoferulic acid 3-*O*-sulfate, ferulic acid 4-*O*-glucuronide, ferulic acid 4-*O*-sulfate, isoferulic acid, isoferulic acid 3-*O*-d-glucuronide, isoferulic acid 3-*O*-sulfate, coumaric acid sulfate*, *m*-coumaric acid, *o*-coumaric acid, *p*-coumaric acid, cinnamic acid, 3,4-dihydroxyhydrocinnamic acid*, dihydroxyhydrocinnamic acid-3-*O*-glucuronide* caffeic acid, caffeic acid 3-*O*-d-glucuronide, caffeic acid 4-*O*-d-glucuronide, dihydro caffeic acid, dihydro caffeic acid 3-*O*-d-glucuronide, dihydro caffeic acid 3-*O*-sulfate, dihydro caffeic acid 4-*O*-sulfate, vanillic acid, vanillic acid-4-*O*-sulfate, homovanillic acid, homovanillic acid sulfate, isovanillic acid, sinapic acid*, sinapic acid glucuronide, syringic acid,	Urine, plasma	Unspecific, common for many fruits and vegetables	[94, 135, 136, 48–51, 174, 175, 183, 27, 7]
	**Flavonols and its derivatives:** kaempferol, kaempferol-3-*O*-d-glucuronide, quercetin, quercetin-3-*O*-d-glucuronide, quercetin 3-galactose, quercetin 3-rhaminoside, quercetin 3-arabinose myricetin, myricetin 3-arabinose, myricetin 3-galactoside epicatechin, isorhamnetin,	Urine, plasma	Unspecific, common for many fruits and vegetables	[135, 136, 48–51, 174, 175, 27]
	**Benzoic acids, and its derivatives:** benzoic acid, 2-hydroxybenzoic acid, 3-hydroxybenzoic acid, 4-hydroxybenzoic acid, 2,3-dihydroxybenzoic acid, 2,4-dihydroxybenzoic acid, 2,5-dihydroxybenzoic acid, trihydroxybenzoic acid, quinic acid, 4-methylgallic acid-3-*O*-sulfate, protocatechuic acid, quinic acid*, vanilloloside*, trihydroxybenzoic acid* hippuric acid, *p*-hydroxyhippuric acid* *m*-hydroxyhippuric acid*, *o*-hydroxyhippuric acid*, alfa-hydroxyhippuric acid*, salicyluric glucuronide*, pyrogallol, pyrogallol-*O*-1-sulfate, pyrogallol-*O*-2-sulfate 1-methylpyrogallol-*O*-sulfate, 2-methylpyrogallol-*O*-sulfate, 3,4-dihydroxybenzaldehyde, 4-hydroxybenzaldehyde catechol sulfate*, 4 -methylcatechol-*O*-sulfate, 3-*O*-Methylcatechin-sulphate*, 3,4-dihydroxyphenyl ethanol sulfate*	Urine, plasma	Unspecific, common for many fruits and vegetables	[94, 135, 136, 48–51, 174, 175, 183, 27, 7]
	**Phenyl acetic-, phenyl propionic acids, and its derivatives:** phenylacetic acid, 3-hydroxyphenylacetic acid, 4-hydroxyphenylacetic acid, 3,4-dihydroxyphenylacetic acid, 4-hydroxy-3-methoxyphenyl acetic acid, hydroxyphenylacetic* 2-(4-hydroxyphenoxy)propionic acid, 3-(hydroxyphenyl)proponic acid*	Urine, plasma	Unspecific, common for many fruits and vegetables	[94, 135, 136, 48–51, 174, 175, 183, 27]
	(4r)-5-(3-hydroxyphenyl)-valerolactone-4-*O*-sulfate, 5-(3′,4′-dihydroxyphenyl)-γ- valerolactone*, 5-(3′,4′-dihydroxyphenyl)-γ-valerolactone-3′-*O*-sulphate*, 5-(3′,4′-dihydroxyphenyl)-γ- valerolactone-4′-*O*-sulphate* 5-(3′,4′,5′-trihydroxyphenyl)-γ-valerolactone-3′-*O*-sulphate*, 4-hydroxy-5-(dihydroxyphenyl)-valeric acid*, 5-(3′,4′-dihydroxyphenyl)-γ- valerolactone-3′-*O*-glucuronide*, 5-(3′,4′-dihydroxyphenyl)-γ-valerolactone-4′-*O*-glucuronide*, 5-(3′,4′-dihydroxyphenyl)-γ-valerolactone*, 5-(trihydroxphenyl)-γ-valerolactone*	Urine, plasma	Unspecific, common for many fruits and vegetables	[135, 136, 48–51, 7, 94]
	chlorogenic acid	Urine	Unspecific, common for many fruits and vegetables and tea	[135, 136, 48–51]
	**Others metabolites**: aconitic acid*, citramalic acid*, dihydroxyquinoline*, glycerol 3-phosphate*, hydroxyoctadecanoic acid*, tyrosine*, vanilloylglycine*	Plasma	Unspecific,	[94]
Blackcurrant	**Anthocyanidins and its derivatives:** cyanidin-3-*O*-rutinoside, cyanidin-3-O-glucoside, cyanidin glucuronide delphinidin 3-*O*-rutinoside, delphinidin 3-*O*-glucoside, delphinidin glucuronide,	Urine, plasma	Unspecific, common for many fruits	[75, 76, 80, 105, 114, 115, 139]
	**Benzoic acids and its derivatives:** benozic acid, dihydroxy benzoic acid sulfate*, gallic acid, protocatechuic acid hippuric acid, 3-hydroxyhippuric acid, 4-hydroxyhippuric acid, catechol sulfate*	Urine, plasma	Unspecific, common for many fruits and vegetables	[164, 80, 75, 76, 139]
	**Cinnamic acids and its derivatives:** dimethoxy cinnamic acid sulfate*, ferulic acid, ferulic acid sulfate* caffeic acid sulfate*	Urine, plasma	Unspecific, common for many fruits and vegetables	[80, 164]
	**Phenylacetic acid and its derivatives:** dihydroxy phenylacetic acid sulfate*	Urine	Unspecific, common for many fruits and vegetables	[164]
	Quercetin	Plasma	Unspecific, common for many fruits and vegetables	[44]
	Vitamin C	Plasma	Unspecific	[80]
	uric acid	Plasma	Unspecific, endogenous compound	[80]

* Metabolites found using untargeted metabolomics

### Application of validation criteria

According to Dragsted et al. [40], a set of criteria was applied on the candidate BFIs reported in Table 1, to assess their current status of validation and identify the missing information for a full validation of each of them.

## Results

### Berry biomarkers

From 852 citations, 75 human studies were selected for this review (Fig. 1), most of them employing a targeted analysis, while only 7 used an untargeted approach [2, 5, 7, 31, 64, 94, 95]. Targeted analyses have mainly quantified anthocyanins in their native glycosidic or aglycone forms, as well as ellagitannins and their microbial metabolites. Some investigations also included quantification of phenolic acids and their conjugates.

After careful application of the inclusion and exclusion criteria to the citations collected with the databases search, the numbers of human studies selected for this review were 22 studies out of 206 for blueberry intake [2, 3, 12, 13, 32, 34, 37, 50, 81–83, 91, 103, 105, 106, 112, 137, 153, 160, 178, 185]; 20 out of 137 for strawberries [4, 5, 9, 14, 20, 22, 26, 31, 41, 48, 74, 75, 113, 126, 129, 141, 147, 148, 165, 179], 7 out of 87 for raspberry [70, 78, 86, 95, 98, 156, 184], 5 out of 66 for blackberries [47, 64, 89, 100, 161], 13 out of 236 for cranberries [7, 27, 49, 51, 94, 108, 119, 127, 135, 174–176, 183], and 8 out of 61 for blackcurrant [44, 76, 80, 105, 114, 115, 139, 164]. No publication on redcurrant could be found relevant for this review.

### Grape biomarkers

From the 880 unique citations screened, 32 original papers fulfilled the inclusion criteria of this review (Fig. 1) [6, 15, 19, 20, 23, 24, 29, 33, 55, 66, 69, 71, 79, 84, 85, 96, 97, 99, 107, 117, 118, 121, 122, 131, 138, 142, 143, 149, 150, 157, 158, 168, 171, 177]. The grape extract was included in this review, considering that elimination of water fraction during desiccation does not drastically change the overall polyphenol content. Most of these studies were nutritional intervention studies with grape juice or grape extract and used a targeted approach to analyze specific metabolites associated with their intake in urine and/or plasma samples. Only few studies used an untargeted metabolomics approach to obtain a holistic view of the metabolites associated with grape intake.

## Discussion

### Berries

Berries, both botanical and aggregate, share several polyphenol families and indeed most of the investigations reported in this review focused on anthocyanins, procyanidins, and other small phenolic metabolites as the main compounds recovered in biological fluids after berry intake. Certain attention was also put on berry aroma compounds. Supplementary Table 1 reports the composition of various berries, highlighting the similarities among them, as well as some characteristic compounds, especially specific aroma compounds, which could potentially be of interest as candidate BFIs.

In the case of anthocyanins, the parent glycosides as well as the corresponding aglycones cyanidin, delphinidin, pelargonidin, malvidin, and peonidin have been analyzed, while their sulfates and glucuronide conjugates were rarely quantified because of the lack of commercial standards. Only four studies included sulfate and glucuronide conjugates of cyanidin, peonidin, delphinidin, and petunidin in the analysis [5, 47, 81, 98]

Ellagitannins were the second family of interest found in the literature related to non-botanical berry consumption. The most frequently reported ellagitannin metabolites included ellagic acid and several microbial metabolites, such as urolithin A, urolithin B, urolithin C, urolithin D, and urolithin M5. Additionally, three studies included glucuronide and sulfate conjugates of urolithins ([25, 26, 98] A,B, [165]). A reason for considering urolithins as putative BFIs for berries is the presence of ellagitanins (from several hundreds of mg/kg to > 1 g/kg) in strawberries, raspberries, and blackberries, with a limited number of other food sources which could interfere.

We examined the relevance of these compounds and others as putative BFIs for every type of berry.

### Blueberries

Eight studies investigated anthocyanin bioavailability after consumption of blueberries in different forms: frozen blueberries, fresh bluberries, blueberry extract, blanched and unblanched blueberries, blueberry juice, and freeze dried berries [37, 81, 82, 103, 105, 112, 178, 185]. The major anthocyanins in blueberries are glycosides of delphinidin and malvidin (Supplementary Table 1). In most studies, the anthocyanin profile in plasma or urine matched the composition of the ingested blueberry. Three malvidin glycosides were the dominant urine anthocyanins in the study by McGhie et al. [105]. Mazza et al. [103] and Del Bo et al. [36] reported malvidin-3-galactoside and malvidin-3-glucoside as the most concentrated metabolites in serum, followed by delphinidin-3-glucoside and petunidin-3-glucoside, this pattern perfectly reflecting the composition of the blueberry extract used in the study. Zhong et al. [185] found delphinidin glucuronide and dephinidin glucoside followed by malvidin glucoside and petunidin glucoside as major metabolites in urine. Unexpectedly, Kalt et al. [81–83] found pelargonidin glucuronide being dominant with delphinidin glucuronide in 24-h urine. As the blueberry juice used was devoid of pelargonidin aglycone or glycosides, the authors suggested that the presence of pelargonidin metabolites may be explained by an interconversion between anthocyanins. Although the detection of anthocyanins may indicate the consumption of certain berries, their low concentrations as well as their chemical instability appear to drastically limit their use as quantitative BFIs, and most likely also qualitative use.

Not only were anthocyanins found in biological fluids, but also a number of phenolic compounds, some of them being anthocyanin microbial metabolites [3, 12, 32, 50, 53, 112, 134, 136, 137, 153, 160]. In the study by Feliciano et al. [50], a total of 61 phenolic metabolites were quantified in plasma at baseline, of which 43 increased after acute or 1-month supplementation with blueberry drinks. Benzoic and catechol derivatives represented more than 80% of the changes in the phenolic profile after 2 h consumption on day 1, whereas hippuric and benzoic derivatives were the main compounds that increased at 0 and 2 h, respectively on day 30. All of these metabolites, however, are common for a wide range of fruits and vegetables, and therefore cannot be considered as specific biomarkers of blueberry consumption.

Two studies used an untargeted approach aiming at exploring the metabolome changes after blueberry intake [2, 91]. Ancillotti et al. reported a high number of plasma and urine metabolites that were statistically significant after consumption of a single 25-g dose of *Vaccinum myrtillus.* The authors applied a robust analysis of MS/MS patterns for a reliable identification. However, they did not find metabolites specific enough for blueberry to serve as candidate BFI. Among the most discriminant metabolites were benzoic, ferulic, and phenylpropionic acid derivatives, as well as phenyl-valeric acid and phenyl-valerolactone derivatives. Additionally, two anthocyanins, delphinidin hexoside and cyanidin hexoside, were detected in urine. Langer et al. similarly attempted to identify blueberry-derived metabolites in plasma and urine metabolomes 2 h after intake of fresh blueberries or juice. However, compound identifications were only putative, without acquisition of MS-MS spectra, and mostly non-specific phenolic acids were reported.

Based on the available studies, it is hard to propose any candidate BFI for blueberry. The presence of malvidin and its phase II metabolites in urine, together with other anthocyanins, may indicate blueberry intake. However, they are obviously not specific and robust enough to constitute a BFI. Another limitation is the very low concentration of these metabolites in biological fluids, frequently below the limit of detection. Further studies should definitely be undertaken in humans, considering other components than anthocyanins in blueberries. Application of untargeted metabolomics with a reliable and robust annotation process, may bring new insights on unforeseen markers of blueberry intake. In particular, the increasing availability of chemical standards, the enrichment of spectral libraries and new bioinformatics tools allowing accurate prediction of metabolism, and MS fragmentation will help in the future to improve the annotation of discriminating compounds, and possibly reveal unforeseen candidate BFIs for blueberry.

### Cranberries

Cranberry is reported to have health benefits, including prevention of urinary tract infections, due to its high content in phytochemicals, including proanthocyadinins A and other polyphenols [54, 88, 125]. Although these health benefits have been recognized, a very limited number of studies have investigated the circulation of cranberry-derived metabolites in humans [7, 27, 49, 94, 108, 119, 175]. Among the publications included in this review, some focused on the analysis of structurally related anthocyanin metabolites [27, 108, 119], which are present in very low amounts in plasma in comparison to their phenolic acid metabolites, while others quantified small phenolic acids after cranberry intake [49, 51, 135, 174, 175, 183]. Interestingly, two studies used an untargeted approach to investigate changes in plasma and urine metabolome after consumption of cranberry juice [7, 94].

Four studies have focused on targeted analysis of anthocyanins and flavonols [27, 108, 119, 175]. Seven anthocyanins were found in plasma at low nanomole per litre levels after consumption of a single dose of 480 mL of cranberry juice, namely peonidin-3-*O*-galactoside > cyanidin-3-*O*-arabinoside > cyanidin-3-*O*-galctoside > peonidin-3-*O*-arabinoside > cyanidin-3-*O*-glucoside > peonidin-3-*O*-glucoside > malvidin-3-*O*-glucoside [108]. The pattern of anthocyanin glucosides observed in urine was similar to that in plasma, with the exception of malvidin-3-*O*-glucoside, and generally reflected the relative concentrations present in the juice or the cranberry extract beverage [27, 108, 119].

One study aimed at verifying whether proanthocyanin dimer A-2 could be a valid biomarker of cranberry intake. A progressive, weekly dosing schedule protocol was applied, involving 5 healthy females drinking for 7 weeks a cranberry juice at increasing frequencies [174]. While proanthocyanin dimer A-2 was quantifiable in urine, it did not appear to be excreted in a concentration that corresponded to the dosing schedule and intake of cranberry juice. Additionally, proanthocyanin dimer A-2 was not detected in all spot and 24-h urine samples of the three subjects. These data suggest that proanthocyanin dimer A-2 cannot be considered as a good candidate intake biomarker. Liu et al. [94] studied metabolome changes in plasma after cranberry juice intake, compared with apple juice intake, assuming that different types of procyanidins (A-type vs B-type, respectively) from the two juices can differently impact the human metabolome. Through the untargeted assay, several endogenous and exogenous metabolites were found to discriminate the two interventions. Among the metabolites differentiating cranberry intake from apple intake were quinic acid, catechol sulfate, 3,4-dihydroxyphenyl ethanol sulfate, coumaric acid sulfate, ferulic acid sulfate, citramalic acid, hydroxyoctadecanoic acid, hippuric acid, 2-hydroxyhippuric acid, and vanilloylglycine (Table 2). Although among the annotated metabolites, none seem specific to cranberry, several discriminant features remained unidentified. Further efforts for more extensive and reliable annotation are required, to ensure full structure elucidation of all highly discriminant metabolites. Baron et al. [7] used a mixed untargeted and targeted approach to analyze urine samples from a randomized, double-blind, 2-arm repeated measure cross-over study with cranberry extract. Again, only non-specific small phenolics and valerolactones conjugated to sulfate and glucuronide moieties were identified among the discrimimant features (Table 2).

A wide range of different classes of phenolic acids and their sulfate and glucuronide conjugates have been quantified in plasma and urine after cranberry intake [49, 51, 127, 135, 175, 183]. Rodriguez-Mateos et al. [135] investigated the absorption, metabolism, and excretion of cranberry polyphenols in the plasma and urine of healthy young men after the consumption of 450 mL of cranberry juice, providing an increasing dose of total polyphenols: 409 mg, 787 mg, 1238 mg, 1534 mg, and 1910 mg. In plasma, hippuric acid and catechol-*O*-sulfate were found at the highest concentrations, followed by 2,3-dihydroxybenzoic acid, phenylacetic acid, and others (see Table 2). The metabolite profile found in urine was very similar, with hippuric acid representing 64% of the excreted metabolites, while 14% were phenylacetic acids, followed by benzoic acids (9%), catechols (5%), valerolactones (4%), and cinnamic acids (3%). Total plasma, but not urinary excreted (poly)phenol metabolites, exhibited a linear dose response, driven by caffeic acid 4-*O*-ß-d-glucuronide, ferulic acid derivatives, and benzoic acid derivatives (see Table 2).

Recently, terpenes have gained considerable attention, being found at significant concentrations in cranberries [87]. Ursolic acid, a ubiquitous triterpenoid, was found as the dominant terpene in American cranberries (46–109 mg/100 g FW), together with two other rare derivatives of ursolic acid: *cis*-3-*O*-*p*-hydroxycinnamoyl ursolic acid (12–16 mg/100 g FW) and trans-3-*O*-*p*-hydroxycinnamoyl ursolic acid (42–60 mg/100 g FW) [87]. Monotropein, 6,7-dihydromonotropein, and other iridoid derivatives have also been described in cranberry [16]. More effort should be put on performance of the untargeted experiments, which have the potential to reveal new BFIs.

### Blackcurrant

Blackcurrant phytochemicals have been found to offer a variety of beneficial effects, including immunomodulatory, antimicrobial, and anti-inflammatory actions and inhibition of low-density lipoprotein, while the high vitamin C content of blackcurrant (18–103 mg/g fresh weight) can help to achieve the recommended daily intake [56, 73, 77, 80, 109, 124, 130]. However, little is known about the recovery of blackcurrant metabolites in biofluids and no attempt to identify BFI has been made so far. Only a few studies have investigated the bioavailability of blackcurrant anthocyanins, which is of little relevance for identifying candidate BFIs. Two anthocyanins, namely delphinidin-3-rutinoside and cyanidin-3-rutinoside were reported in urine samples collected 2–4 h postprandially ([76, 115]; and [80]. Only one study reported anthocyanins in plasma Nakamura et al. [114] quantified the following compounds in decreasing order of concentration: delphinidin-3-rutinoside > cyanidin-3-rutinoside > delphinidin-3-glucoside > cyanidin-3-glucoside, which was consistent with the respective quantities of the ingested anthocyanins (see Table 1 for details). Blackurrants have an interesting aroma profile with monoterpenoids being particularly high such as 3-carene, sabinene, humulene, α pinene, and terpinolene [17, 39, 52] (see Supplementary Table 1). However, there are no investigations yet on the metabolism of aroma blackcurrant in humans. There is thus a critical need for data related to the impact of blackcurrant on the human metabolome, and for identification of blackcurrant metabolites that could act as potential biomarkers of intake.

### Strawberries

Of the twenty investigations included for this review for strawberry, most used a targeted metabolomics approach to detect the low concentrations of anthocyanins in biological fluids, with the exception of one study [31], where an untargeted approach was used. As regards strawberry composition, pelargonidin-based anthocyanins and procyanidins are particularly high (see Supplementary Table 1) but not specific to strawberry, while furaneol and mesifurane are characteristic aroma compounds of strawberry.

Several studies reported pelargonidin metabolites in the urine metabolic profile after strawberry intake [4, 20, 22, 113, 126]. Mullen et al. identified four pelargonidin glucuronide isomers, pelargonidin-3-*O*-glucoside and pelargonidin aglycone in plasma and urine, while pelargonidin sulfate was detected only in urine. Similar profiles were found in urine by Carkeet et al. [22], Azzini et al. [4], Park et al. [126], Felgines et al. [48], Hollands et al. [75], and [20]. Three investigations considered dose–response effects [22, 48, 75] and reported that urinary excretion of pelargonidin-3-glucoside and pelargonidin glucuronide isomers increased linearly with the dose of strawberry. However, the total urinary excretion of strawberry anthocyanin metabolites remained low corresponding to only 0.9–1.80% of the pelargonidin-3-glucoside ingested.

The profiles of pelargonidin metabolites in plasma were similar to those in urine [5, 41, 113, 179]. Banaszewski et al. [5] characterized the postprandial plasma profile of strawberry anthocyanins, particularly pelargonidin-based anthocyanins, across 4 doses using targeted and untargeted analysis. Pelargonidin-*O*-glucuronide was found to be the primary pelargonidin metabolite and showed a dose–response relationship. However, while the maximum concentration in plasma and the area under the concentration–time curve increased with the dose, the concentrations decreased as the percentage of the dose, suggesting a possible saturation of the absorptive mechanisms or an increased efficiency in elimination. Apart from the glucuronide conjugate, qualitative Q-TOF LC/MS analysis revealed 33 other phenolic compounds, including a wide range of pelargonidin metabolites, such as pelargonidin sulfate, pelargonidin-3-*O*-6′′-rhamnosylglucoside, carboxypyranopelargonidin-3-*O*-glucopyranoside, pelargonidin-3-(6′′-caffeoylglucoside), pelargonidin-3,5-diglucoside, pelargonidin-3-sambubioside, and pelargonidin-3-(6′′-malonylglucoside). Similarly, Edirisinghe et al. [41], who analyzed plasma using targeted LC-MS/MS, detected pelargonidin sulfate, pelargonidin-3-*O*-glucoside, and pelargonidin-3-*O*-glucuronide to be the most abundant metabolites following intake of a single portion of strawberries. Quantification of pelargonidin sulfate, pelargonidin glucoside and pelargonidin glucuronide showed concentrations in the low nanomole per litre range, with T_max_ varying between 1.1 and 2 h [41, 113, 147].

Urolithins have been the subject of investigations by several authors [9, 14, 25, 26, 129, 148, 165], and their appearance in biological fluids was studied after strawberry intake in chronic and acute modes. In an acute study [25], the microbial metabolite urolithin B glucuronide was first detected along the urine fractions from 32 to 56 h. Considerable inter-individual differences, probably associated with differences in colonic microbiota composition, were noted, identifying “high- and low-metabolite excreters”. Later, Truchado et al. [165] found urolithins in urine profiles after fresh strawberry intake, with urolithin A glucuronide as the predominant metabolite, followed by urolithin A, urolithin B, and urolithin B glucuronide. All urolithins appeared in urine within a time ranging from 8 to 32 h. Urolithin B and urolithin B glucuronide were detected in only 4 volunteers (20% of the total population), consistently with the reported fact that only part of the population synthesizes urolithin B; actually, only part of the population has the microbiota species that are able to realize all metabolic steps required to convert ellagitannins/ellagic acid into urolithin B. The mean urinary excretion of urolithin conjugates over 92 h reached 58 ± 48% of the amount of total ellagic acid administered (62 mg) after fresh strawberries and 57 ± 52% after strawberry puree. On the other hand, no urolithin A was found in plasma after several days of strawberry consumption in two studies [14, 129], and only trace amounts of ellagic acid (15.2 ± 5.2 ng/mL) were detected by Basu et al. [9]. Urolithins present several limitations as candidate BFIs. Walnuts and other fruits contain ellagitannins; thus, the specificity for strawberry is quite low, although strawberries are a very rich source of both simple and complex ellagitannins [67], and among the fruit containing ellagitannins, strawberries are the most widely consumed. Their main ellagitannin, agrimoniin, has been suggested to be one of the most widely present ellagitannins in the human diet due to strawberry intake [172], while casuarictin is the second characteristic strawberry ellagitannin [67]. The interindividual variation in urolithin production by the microbiota is also likely to affect their accuracy as quantitative BFIs. However, the interest of total urolithins as components of a multi-marker BFI for strawberries deserve further investigation, especially in observational setting.

Three studies suggested the importance of strawberry aroma compounds in the search for candidate BFIs. One untargeted experiment was performed by Cuparencu et al. [31], in which three furaneol and mesifurane metabolites were found in urine, namely furaneol glucuronide, furaneol sulfate, and mesifurane sulfate. This randomized controlled crossover meal study was designed to study urine kinetics after a single intake of 150 g of strawberry puree. Furaneol metabolites had already been the subject of investigation in 1997 by Roscher et al. [141], who reported furaneol glucuronide to be the main strawberry aroma metabolite in urine. In 2010, Henning et al. [74] quantified furaneol glucuronide using LC-MS/MS in both plasma and urine of 21 volunteers receiving 250 g of strawberries for 3 weeks. Plasma concentrations at 6 h were 148.9 ± 223.8 nM, while in 0–12-h urine, 14.5 μmol/g creatinine were detected. Although fairly specific for strawberry, aroma metabolites have still not gained much attention as intake biomarkers. Furaneol was also found in pineapples, raspberries, or kiwi fruits; moreover, it is a product of Maillard reactions [151], which indicate cooking processes such as baking or frying. Therefore furaneol derivatives alone can hardly be proposed as BFI for strawberry intake.

**Fig. 2:**
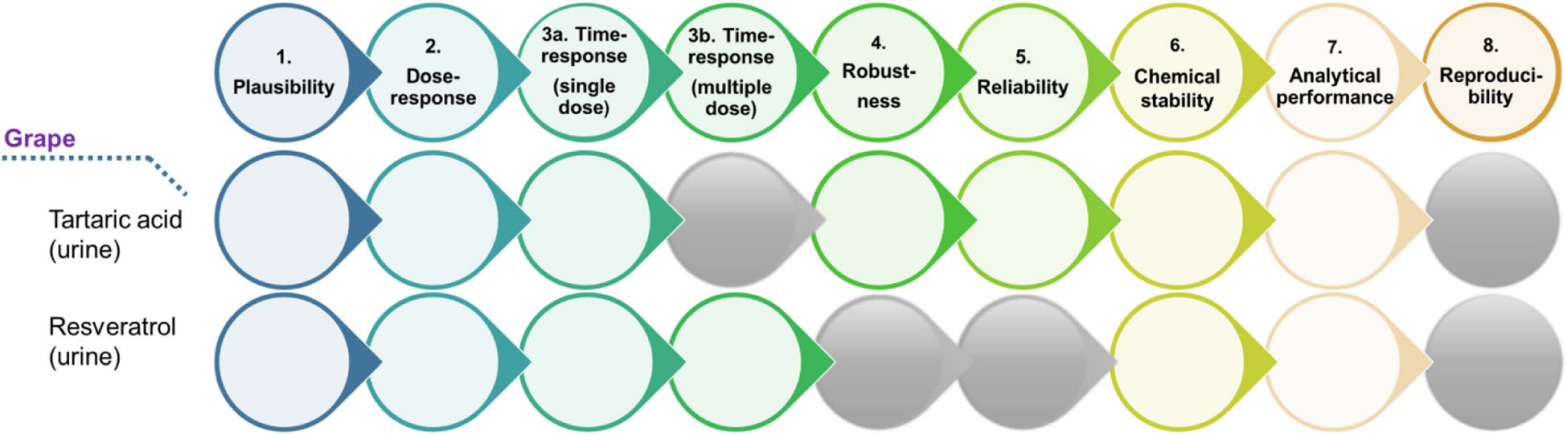
Overview of the validation process and its application on candidate BFIs for grape

In terms of biomarker validation criteria, pelargonidin and its conjugates, as well as utolithins achieve plausibility criterion, however fail at specificity. Aroma metabolites seem to be plausible and specific; however, there is a lack of information related to the concentration in biological fluids. Definitely more studies are required to understand ADME processes of aroma compounds in humans. To summarize, based on current knowledge, only a multi-metabolite biomarker panel could be suggested to indicate strawberry intake (see Fig. 2 with validation scheme). Pelargonidin glucuronide together with urolithins and furaneol/mesifuran derivatives seem the most appropriate candidates; however, differences in excretion rates requires an intensive work to identify the most applicable biofluid and time for sample collection.

### Raspberries

Raspberries have a phytochemical profile also rich in ellagitannins and anthocyanins, with sanguiin H-6 and lambertianin C, as well as cyanidin-3-*O*-sophoroside, cyanidin-3-*O*-(2-*O*-glucosyl)rutinoside and cyanidin-3-*O*-glucoside, being the most characteristic compounds. The specific aroma of raspberries is mainly due to the presence of 4-(*p*-hydroxyphenyl)-2-butanone, commonly called raspberry ketone.

There are no human studies considering the metabolism of raspberry aroma compounds, although such compounds seem to be very specific. The metabolism of raspberry ketones has been investigated in animals only: rats, guinea pigs, and rabbits [156]. Raspberry ketone was rapidly absorbed from the gastrointestinal tract after administration of a single dose of 1 mmol/kg (164 mg/kg bw) by oral gavage. The majority of ingested raspberry ketone was excreted in urine within the first 24 h as its phase II conjugated metabolites (glucuronide and sulfate forms). In addition to raspberry ketone phase II metabolites, a total of 14 other compounds were detected in urine. Of these, the reduction product 4-(4-hydroxyphenyl)butan-2-ol was the most abundant, followed by 4-hydroxyphenylacetic acid, 2-(4-hydroxyphenyl)ethanol, 4-(3,4-dihydroxyphenyl)butan-2-one, and 4-(4-hydroxyphenyl)butan-2,3-diol. Total urinary recovery was close to 90% for the three animal species. Considering that raspberry ketone is specific to the raspberry and seems to be well absorbed, more effort is obviously required in the future to determine and characterize the metabolites of this particular aroma compound in human biological fluids. However, raspberry ketone is increasingly used as a food additive due to its pleasant flavour. Thus, its detection would have to be accompanied by other raspberry metabolites to reflect berry intake and not consumption of aromatized foods.

Metabolites of raspberry ellagitannins and anthocyanins have been the focus of investigations by several groups [70, 78, 98, 184]. In the study by Ludwig et al. [98], a total of 27 metabolites were detected in urine for over a 48-h collection period after a single intake of 300 g of raspberry, and 15 metabolites were found in plasma for over a 24-h collection period. Cumulative urine metabolite profile was dominated by derivatives of phenylpropanoic acid, urolithins, and ellagic acid, while mainly urolithins and ellagic acid derivatives were detected in plasma. The profiles of urolithin metabolites in both matrices were very similar, with urolithin A-sulfate as the dominant metabolite, followed by urolithin A-*O*-glucuronide, urolithin B-3-*O*-glucuronide, (iso)urolithin A-sulfate-*O*-glucuronide, isourolithin A-*O*-glucuronide, dimethylellagic acid-*O*-glucuronide, characterized by T_max_ between 8 and 24 h and concentrations ranging between 3.5 and 450 nmol/L. The presence of cyanidin-3-*O*-glucoside in urine and plasma after raspberry intake was found at low nanomoles per litre levels by the three authors (González-Barrio et al. [70], Ludwig et al. [98], and Istas et al. [78]), confirming that native anthocyanins occur only at low trace amounts in biological fluids.

The other metabolites reported in urine and plasma after raspberry intake included mainly small phenolic acids belonging to benzoic, phenyl acetic, and hippuric acid derivatives, as well as ferulic and caffeic acid derivatives, ascorbic acid, and methylepicatechin sulfate. All these metabolites, however, occur frequently in urine after different nutritional interventions with plant foods, therefore cannot be considered as candidate BFIs for raspberry even as part of a multi-marker [78, 95, 98, 184].

There is a considerable need for more human studies focusing on raspberry intake. It might be speculated that raspberry ketone metabolites together with total urolithin metabolites in urine samples could be considered as potential candidates of indicative markers, or act as multi-metabolite biomarker panel; however, the topic remains widely open.

### Blackberries

In the case of blackberries, investigations included in this review also mostly considered metabolites of anthocyanins and ellagitanins. Three studies were single-dose experiments involving healthy volunteers [47, 64, 100], and in all of them, a metabolomics approach targeted on cyanidins and urolithins was applied for plasma and urine profiling.

Cyanidin metabolites were found in biological fluids by Felgines et al. [47], García-Muñoz et al. [64, 65], and Marques et al. [100]. In Marques’ study, methyl cyanidin glucuronide and 3’methyl-cyanidin-3-glucoside were the main anthocyanin conjugates detected in both plasma and urine samples. Apart from these metabolites, other minor compounds were detected: cyanidin glucuronide, cyanidin glucoside sulfate, methyl cyanidin glucoside, cyanidin-3-glucoside-7-glucuronide, cyanidin rutinoside, and cyanidin-3-glucoside. For the metabolites mentioned above, T_max_ varied between 66 and 120 min, with C_max_ concentrations between 11 and 272 ng/mL. An investigation by Felgines et al. [47] and Tian et al. [161] confirmed the presence of cyanidin monoglucuronide and the low urinary excretion of native cyanidins such as cyanidin-3-rutinoside, cyanidin-3-glucoside, cyaniding sambubioside, and cyanidin-3-xylosylrutinoside.

García-Muñoz et al. [64, 65] studied ellagitannin metabolism in urine after the ingestion of 250 mL blackberry juice. It is known that the composition in ellagitannins and ellagic acid derivatives is very similar for blackberry and raspberry, as for all Rubus fruits [68]. The primary ellagitannin derivatives found in human urine were urolithin A glucuronide and urolithin B glucuronide, followed by urolithin C glucuronide, urolithin D glucuronide, urolithins A, B, C, D, M5, methyl urolithin A, ellagic acid, ellagic acid dimethyl ether glucuronide, ellagic acid dimethyl ether, and hexahydroxydiphenyl. However, the urolithin metabolite profiles varied considerably between volunteers, as already reported for other dietary sources of ellagitannins, as well as by Kresty et al. [89], who reported that urolithin A glucuronide and urolithin A-sulfate were detected in 85% and 60% of patients, respectively, following 12 and 26 weeks of lyophilized blackberry consumption. The fact that individuals do not respond in the same way after the ingestion of the same food complicates the interpretation of a measured value in terms of dose–response relationships.

Again, more studies in humans are required in order to identify the most specific metabolites for blackberry and better describe their kinetics in plasma and urine. Based on the aforementioned studies, it is difficult to speculate about which metabolites may be potential biomarkers of blackberry intake, while combination of urolithin metabolites in 24-h urine with cyanidin metabolites could only be a rough indicative marker for blackberry consumption, for the reasons already discussed for the other berries.

Given the high similarity of their qualitative composition, the Rubus berries could be evaluated as a combined group. A distinctive feature of the ellagitannins in the Rubus fruit is that they contain the sanguisorboyl group, a relatively uncommon ester between a galloyl group and a (*S*)-hexahydroxydiphenoyl moiety, which is positional isomeric as compared with the more widespread valoneoyl ester group. This building block is present at a concentration close to 50% that of the ellagic acid [173]. Unfortunately, we are currently lacking data about the fate of this structure upon ingestion.

### Berries considered as one food type

As showed above for various berry types, it is not easy to propose candidate BFIs. In most cases, berry-derived metabolites failed the specificity criterion. On the other hand, berries could be considered as a food group, and a panel of metabolites could indicate the consumption of berries without specification of the berry species. In such case, urolithins and anthocyanin metabolites, when present simultaneously in biological fluids, appear as the most relevant candidates. However, several strong limitations exist. One is the high individual variation in the production of urolithins, described with the three metabotypes A, B, and 0 [63, 162, 163]. A way to overcome this issue would be to wisely sum the concentrations of the major urolithins reported in the different metabotypes: urolithin A, urolithin B, urolithin C, and urolithin D, together with their glucuronide and sulfate conjugates [163]. Urolithins are produced by the opening and decarboxylation of one of the lactone rings of ellagic acid and the sequential removal of hydroxyl groups. The decarboxylation step leads first to formation of urolithin M-5, and from this urolithin D, urolithin M-6 through dehydroxylation at different positions. Further dihydroxylation lead to formation of three hydroxy-urolithins: urolithin C, urolithin M-7, and two hydroxy-urolithins: urolithin A and isourolithin A. Finally the monohydroxy urolithins are urolithin B and isourolithin B [45]. A number of studies investigating metabolism of ellagitannins reported urolithin A and B glucuronide and sulfate, as the main metabolites in urine and plasma, while urolithin C and isourolithin A glucuronide were minor metabolites [28, 65, 72, 92, 140, 165]. Considering anthocyanins, the following metabolites were the most frequently found in urine after different berry consumption: cyanidin glycosides, cyanidin glucuronide, malvidin glycosides, malvidin glucuronide, pelargonidin glycosides, and pelargonidin glucuronide. Delphinidin glycosides and glucuronide together with petunidin metabolites could be considered as minor metabolites. An appropriate collection of samples for both anthocyanins and urolithins should be carefully evaluated and may be difficult to implement, as these compounds have markedly different pharmacokinetics. Urolithins appear in plasma and urine for only several hours (5 h and more) after an ellagitannin-containing meal, and are still detectable around 12–36 h [30], while anthocyanins have a T_max_ of 1–2 h [30]. Then intensive investigations are required to verify whether such multi-metabolite panel could accurately indicate consumption of berries in large populations. Another important issue to consider is the presence of confounding factors such as consumption of other foods rich in ellagitanins and anthocyanins. Special attention should be put on nut intake, as these are a well-known source of ellagitannins [58]. Regarding anthocyanin intake, among vegetables and fruits, red radish, red cabbage, black beans, eggplants, grapes, pomegranate, and cherries represent likely confounding factors [104].

### Grapes

Among the studies selected for this review, only a few applied an untargeted metabolomics approach to obtain a holistic view of the metabolites associated with the intake of grape or grape extracts [66, 85, 168]. Garcia-Perez et al. aimed to identify BFI and proposed a pipeline method using grape as an example. The method was based on the combination of an untargeted discovery approach in an acute study followed by a hypothesis-driven targeted analysis in a short-term dietary intervention. Using NMR, the authors first identified tartaric acid as the most discriminating metabolite in urine after red grape intake, while in a second step a targeted approach was used for quantification of this acid in an independent dietary intervention (Table 1). In the discovery study, the authors observed that other NMR signals from glucose, hippurate, and 4-hydroxyhippurate were also associated with grape intake; however, only tartaric acid increased proportionally in all participants with incremental grape intake over the duration of the study. Then they confirmed with a targeted analysis that tartaric acid excretion was elevated in 24-h urine samples after 4 days of consumption of grapes, reaching a level accounting for 19% of the dose ingested from grapes. The relationship was dose-dependent as after consumption of 50, 100, and 150 g of grapes, an average amount of 0.16, 0.30, and 0.49 mmol of tartaric acid, respectively, was excreted in 24-h urine [66]. This observation complemented the results previously published by Lord et al. [97] which showed an increase in urinary tartrate excretion from 7.4 to 282 μg/mg of creatinine after an acute intake of 280 mL (10 oz) of grape juice and by Stalmach et al. [158] who reported excretion of 59±14 μmol over 24 h after intake of 350 mL of Concord grape. Some pharmacokinetics data are available for tartaric acid, showing that its excretion peaks between 4 and 8 h after grape intake, with a majority of the excretion occurring in the first 12 h [66].

Two other studies applied an untargeted metabolomics approach providing information on the changes occurring in 24-h urine fingerprints after an intervention with grape extracts for 2 or 4 weeks. In the first study on 58 subjects [168], authors showed differences in 18 metabolites detected by gas chromatography–mass spectrometry (GC-MS), corresponding to metabolites derived from host and gut microbiota metabolism of grape phenolic acids (Table 2). The other study was developed on 31 subjects after an acute or a 15-day supplementation with a drink containing grape skin extract [85]. The authors pointed out that the postprandial urinary metabolome determined by LC-MS after a single dose was characterized by phase II metabolites of polyphenols present in grape skin, whereas in 24-h urine samples collected after a prolonged consumption, phase II metabolites derived from microbial metabolism of flavanols were predominating (Table 2). However, the identified metabolites do not have a high specificity for grape consumption, since they were also detected in urine after consumption of other polyphenol-rich sources, such as cocoa, tea, red wine, or almond [144].

In term of validation of tartaric acid as BFI, the plausibility criterion seems fulfilled, as tartaric acid was described as the main organic acid in red and green grapes [66], as well as in wine [133]. Tartaric acid biosynthesis begins with L-ascorbic acid through the cleavage of a six-carbon intermediate between position C4/C5. This is the predominate pathway in the *Vitaceae* plants [35, 146]. On the other hand, it can be present in some processed foods, such as mixed dishes (0.42 g/kg), biscuits (0.12–0.13 g/kg) or chocolate (0.00–0.18 g/kg), where it is added as an acidifying agent. However, concentrations reported in these dietary sources were markedly lower than in grapes (6–9 g/kg) and wine [11, 155]. Actually, tartaric acid has also been reported in urine after wine intake [132, 169]. Consequently, tartaric acid could be considered as a candidate biomarker, not just for grapes but also for all grape-derived products including wine. The analytical performance, as well as chemical stability were proved in both targeted and untargeted experiments. Dose–response experiments confirm this validation criterion, while time–response for multiple doses and robustness in observational studies still have to be evaluated; see Fig. 2.

Beside tartaric acid, resveratrol metabolites can be considered as relevant candidate BFI for grape. Resveratrol is a natural stilbene mainly present in grapes and its derivatives including juices and wine. Being mainly extracted from the skins, where it is present both in the free forms (*cis* and *trans*) and as their 3-glucosides (piceid) [101], resveratrol presence is higher in whole grapes and in red wines than in white wines and juices. Rotches-Ribalta et al. [143] provided a comprehensive overview of the resveratrol metabolic profile. The metabolism of resveratrol in humans involves the formation of glucuronides and sulfate conjugates, as well as conjugates of dihydroresveratrol (DHR), which is a metabolite derived from the hydrogenation of resveratrol by the action of the gut microbiota. Four studies showed increased levels of resveratrol and its derived metabolites after grape juice or grape extract consumption, mainly through acute studies [107, 121, 142, 143] (Table 1). Meng et al. [107] were not able to detect resveratrol in hydrolysed urine samples after the intake of 200 and 400 mL of grape juice, but its level was increased after doses of 600 and 1200 mL, which suggests a dose–response relationship. Some of these studies provided pharmacokinetics data for resveratrol after grape intake. In one of them, after consumption of 1 L of grape juice, plasma concentrations of *cis*- and *trans*-resveratrol reached maximal levels (36.5 ng/mL in hydrolysed samples, 0.16 μM) 4 h after grape juice. Then their levels gradually decreased throughout the next 12 h and were no longer detectable in some subjects after 24 h [121]. Rotches-Ribalta et al. [142] also observed in non-hydrolysed samples that although resveratrol glucosides (piceid) can be detected in plasma as intact forms, they reached maximum concentrations (4.5 ng/mL) 1 h after an intake of grape extract tablets, whereas after about 4 h, much higher plasma concentrations (14.83–35.23 ng/mL) were observed for resveratrol glucuronides and after 7–8 h, even higher concentrations for DHR glucuronides (23.06–85.70 ng/mL). Plasma levels of resveratrol glucuronides showed two maximum peaks (at 4 h and 8 h), suggesting an enterohepatic recycling [142]. Whereas only glucuronide conjugates were found in plasma samples; in urine samples, both glucuronide and sulfate conjugates were detected [142, 143]. In parallel with the plasma results, the highest excretions of DHR metabolites were achieved later than resveratrol phase II metabolites and up to 24 h after the intake of grape extract [142, 143]. On the other hand, Ortuño et al. [121] observed that the urinary excretion of resveratrol isomers increased after the consumption of grape juice, reaching a peak at 0–4 h with a recovery of 14.2 ± 5.9% of the dose (0.6 ± 0.3 μmol). Surprisingly, the microbial metabolite DHR was not detected in urine after grape intake, although in the same study, an increase of its urinary excretion was observed after wine consumption. It is important to keep in mind that a high variability in the amounts of resveratrol excreted in urine was observed within subjects, and this variability was even more important for microbial metabolites [142, 143]. In addition, the proportion of the various resveratrol metabolites may depend on the dose, with a higher proportion of sulfates compared to glucuronides when the ingested dose increases [142]. These aspects must be considered when formulating a quantitative threshold from which we could confirm that a person has ingested grape or not.

As regard to BFI validation (Fig. 2), the first plausibility criterion for resveratrol is fulfilled, as wines and grapes are the major dietary sources [181], although it is also present in other foods, such as apple, peanuts, pistachios, or berries. According to Farneti et al. [46], apples can be a possible source of dietary intake of resveratrol; however, resveratrol or its metabolites were not found so far in biological fluids after apple intake. Regarding the analytical performance for determining resveratrol metabolites in biological samples, Urpi-Sarda et al. [167] reported that resveratrol and its metabolites were stable in urine samples from subjects who consumed red wine under different storage and sample handling conditions, after freeze and thaw cycles and short- and long-term (5 years) storages. In parallel, quantification methods of these metabolites in biofluids showed good analytical validation parameters (ie., analytical variability, accuracy, sensitivity, specificity, precision, limits of detection, and quantification) [121, 142]. In summary, the use of the sum of resveratrol metabolites as BFI of grape might be suitable because of the significant increase after grape consumption, the specificity for this food and a known kinetics during the 24-h following the ingestion. However, it would be highly recommended to develop further studies to evaluate more in depth the dose–response relationship and the inter-individual variation, especially for microbial metabolites. This will help to define a specific cutoff value or calibration curve to determine consumption levels, as it has been proposed for wine [182]. As the concentrations of resveratrol metabolites are rather low even after high doses, the minimum intake level measurable will have to be determined.

Except for tartaric acid and resveratrol, no other promising candidate BFI were retained for grapes. Many other studies with a targeted approach focused on the non-specific major polyphenols of grape, namely anthocyanins [15, 55, 157], catechins, and procyanidins [99, 149, 150, 157]. Other non-specific small phenolics, including a range of hydroxybenzoic acids (gallic acid, dihydroxybenzoic acid, vanillic acid, hydroxyhippuric acid, etc.), pyrogallol metabolites, hydroxyphenylacetic acids, or hydroxyphenylpropanoic acids [158, 177] which are either present in the grape or originate from gut-microbiota metabolism have been reported in biofluids after grape intake. The list of these non-specific metabolites is reported in Table 2.

Among anthocyanidins, cyanidin, delphinidin, malvidin, peonidin, petunidin [19, 55, 157], and their glucuronide conjugates [157] were identified in urine, plasma and/or ileal fluid after an acute intake of red grape juice (Table 2). However, anthocyanins are quite unstable compounds, and low bioavailability rates were found in all studies, with maximum detected levels found in the first hour after the intake of the test product and a very rapid elimination. Additionally, these metabolites have been reported after the intake of other anthocyanin-rich foods, such as berries [15, 19, 55, 157]. All of these aspects obviously limit the usefulness of the anthocyanin-metabolites as biomarkers of grape intake, see Table 2.

Increased levels of a wide range of flavan-3-ols metabolites have also been observed (Table 2). Vinson et al. [171] and Lutz et al. [99] reported increased levels of epicatechin and catechin, determined in hydrolysed plasma and urine samples after intake of red grape seed extract and grape juice, respectively. Other reported metabolites are phase II conjugated forms of monomeric flavan-3-ols [157], as well as microbial-derived metabolites [158]. Sano et al. [149] detected procyanidin B1 in its intact form at low levels (nmol range) in human serum 2 h after intake of grape seed extract. However, as previously pointed out, these metabolites do not have enough specificity when evaluated individually since they can also increase after the intake of other flavan-3-ol food sources.

Another group of metabolites widely reported in the reviewed studies is that related to gallic acid. Some authors reported an increased excretion of this hydroxybenzoic acid [79, 99, 157] or its metabolites methyl-gallic acid [131, 177] and pyrogallol [131, 158, 168] after grape consumption. Gallic acid can originate from the food itself or from the degradation of precursor compounds, in particular from the cleavage of galloylated monomeric flavan-3-ols by microbial catabolism (Monagas et al. [111]). Therefore, even if gallic acid metabolites are probably systematically present in biofluids after grape intake, they do not appear as valuable candidate BFIs for grape, if used alone, due to the major confounding of tea for example. Increased levels of quercetin and related-metabolites have also been reported in different studies after grape intake [24, 29, 33, 107], but these metabolites also increase after the intake of onions and tea, among other foods [144].

In light of these results, we can conclude that both tartaric acid and the sum of resveratrol metabolites, understood as glucuronide and sulfate conjugates, appear as the best candidate BFIs available for grapes. However, they could also reflect wine consumption. In this regard, Vázquez-Fresno et al. [169] proposed to use the combination of tartaric acid and ethyl glucuronide to monitor wine consumption. Therefore, this “tartrate-ethyl glucuronide” model could be implemented to differentiate grape intake from wine consumption.

## Conclusion

The results yielded by the bibliographic survey on grape show the complexity lying on finding a unique biomarker of grape intake. Although many metabolites are strongly related to the consumption of this fruit, their variable presence in the matrix, their insufficient specificity and low bioavailability after the ingestion can limit their usefulness as a reliable biomarker of intake. Here, we suggest that the metabolites that could overcome these limitations are tartaric acid and the sum of resveratrol metabolites, associated with the non-detection of ethyl glucuronide or another alcohol biomarker as a confirmation that the detection of tartaric acid and resveratrol metabolites is due to a consumption of grapes as fruit and not as wine.

With regards to berries, considering the metabolites found in biological fluids and reported in this review, no metabolite can be suggested as a specific BFI for any particular berry. Berries share some phytochemicals such as anthocyanins and ellagitannins with other fruits or nuts, and therefore, such metabolites fail the specificity criterion—the first to be verified across the BFI validation system.

**Fig. 3:**
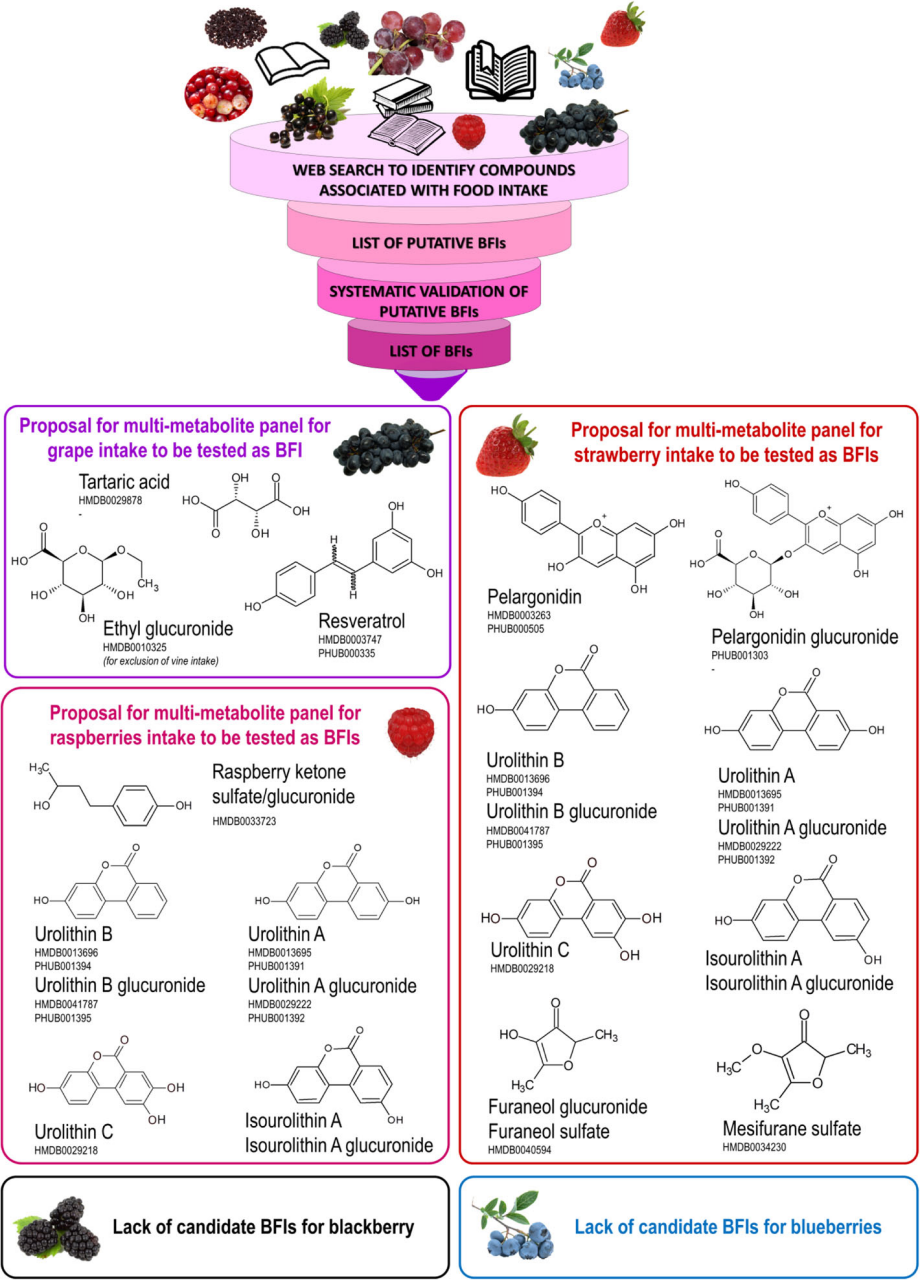
Scheme of literature evaluation process for grapes and berries with BFIs and potential indicative metabolites

Regarding single berry types, a multi-metabolite biomarker panel could be proposed, to evaluate whether a combination of more than one metabolite has a sufficient predictive capacity. In such a way, urolithins derived from ellagic acid and ellagitannins, especially important for strawberry (and Rubus) fruits, together with pelargonidin metabolites and specific aroma compounds such as furaneol/ mesifurane conjugated to sulfate or glucuronides moieties could be associated with strawberry intake. Correspondingly, urolithins together with raspberry ketone metabolites could be associated with raspberry intake. The human metabolism of aroma compounds, especially furaneol, mesofurane for strawberries, or raspberry ketones for raspberries require more investigations regarding dose–response relationship, quantification levels, and possible interindividual variability. Profiling of volatile food compounds is gaining more and more interest, not only in field of food quality and food safety, and could be of relevance for the discovery of novel specific BFIs for berries. A tentative proposal of multi-metabolite panels reflecting specific berry intake is shown in Fig. 3. It is clear that all of them require additional analysis and validation before they can be considered applicable BFIs for berries.

Of the 104 studies reported in this review, only seven applied an untargeted metabolomics approach, and fewer had the objective to identify specific BFIs for berries. Despite the fact that considerable progress has been made in the understanding of the metabolism of berry phytochemicals in humans, there are still important gaps in our knowledge on the ADME in humans of components other than anthocyanins and ellagitannins. In particular, aroma compounds definitely require further attention. Untargeted metabolomics in controlled intervention studies will be an approach of choice for making progress in this field, provided that important efforts are devoted to identify the discriminating metabolites. Metabolite identification remains the bottleneck of biomarker discovery studies applying metabolomics. A profound knowledge of the detailed composition of foods and food products is absolutely required to find a link between metabolites circulating in biological fluids and the food constituents from which they derive. Furthermore, the use of bioinformatics tools BioTransformer (http://biotransformer.ca/) [38], Meteor Nexus (Lhasa Limited, UK), or ADMET Predictor (Simulation Plus, Lancaster, CA, USA) for in silico prediction of metabolism and for supporting the interpretation of analytical data can greatly facilitate that arduous step of metabolite identification.

Finally, as it becomes more and more evident than specific simple biomarkers will not be found for every type of food, complex modelization systems will have to be developed to allow the correct interpretation of combinations of multimetabolites biomarkers, taking into account the possible intake of confounders.

## Supplementary information

**Additional file 1.**

## Data Availability

Not applicable
