# Supplementary material for: Food intake biomarkers for berries and grapes
Source: Genes Nutr. 2020 Sep 23;15:17. doi: 10.1186/s12263-020-00675-z (PMC7509942; doi:10.1186/s12263-020-00675-z)
Supplement: Supplementary file 1 — Additional file 1. [file 12263_2020_675_MOESM1_ESM.pdf]

Table. Main components of berries, concentration expressed in mg/100 g FW (phenol-explorer.eu); in bold – highly concentrated or specific compounds, *mg/kg of fresh weight

Blackcurrant		Strawberry		Blueberry		Raspberry		Blackberry		Cranberry	
Anthocyanins											
Flavonoids											
Cyanidin 3-O-(6''-p-coumaroyl-glucoside)	1.49 mg	Cyanidin	0.50 mg	Cyanidin 3-O-(6''-acetyl-galactoside)	0.10 mg	Cyanidin	0.53 mg	Cyanidin 3-O-(6''-dioxalyl-glucoside)	10.97 mg	**Cyanidin 3-O-arabinoside**	**4.47 mg**
**Cyanidin 3-O-glucoside**	**25.07 mg**	Cyanidin 3-O-(6''-succinyl-glucoside)	1.22 mg	Cyanidin 3-O- (6''-acetyl-glucoside)	0.95 mg	**Cyanidin 3-O-glucoside**	**14.89 mg**	Cyanidin 3-O-(6''-malonyl-glucoside)	4.30 mg	**Cyanidin 3-O-galactoside**	**8.89 mg**
**Cyanidin 3-O-rutinoside**	**160.78 mg**	Cyanidin 3-O-glucoside	2.88 mg	Cyanidin 3-O-arabinoside	2.16 mg	Cyanidin 3-O-glucosyl-rutinoside	7.06 mg	**Cyanidin 3-O-glucoside**	**138.7 mg**	Cyanidin 3-O-glucoside	0.74 mg
**Delphinidin 3-O-glucoside**	**86.68 mg**	Pelargonidin	4.31 mg	Cyanidin 3-O-galactoside	5.41 mg	Cyanidin 3-O-rutinoside	5.20 mg	Cyanidin 3-O-rutinoside	8.86 mg	Peonidin 3-O-arabinoside	9.61 mg
**Delphinidin 3-O-rutinoside**	**304.91 mg**	Pelargonidin 3-O-(6''-malonyl-glucoside)	4.78 mg	Cyanidin 3-O-glucoside	1.37 mg	**Cyanidin 3-O-sophoroside**	**37.61 mg**	Cyanidin 3-O-xyloside	9.74 mg	**Peonidin 3-O-galactoside**	**22.02 mg**
Pelargonidin 3-O-rutinoside	2.48 mg	**Pelargonidin 3-O-(6''-succinyl-glucoside)**	**10.44 mg**	Delphinidin 3-O-(6''-acetyl-galactoside)	0.72 mg	Delphinidin 3-O-glucoside	0.21 mg			Peonidin 3-O-glucoside	4.16 mg
Peonidin 3-O-rutinoside	1.27 mg	Pelargonidin 3-O-arabinoside	0.42 mg	Delphinidin 3-O-(6''-acetyl-glucoside)	2.39 mg	Malvidin 3-O-glucoside	0.62 mg				
Petunidin 3-O-(6''-p-coumaroyl-glucoside)	5.45 mg	**Pelargonidin 3-O-glucoside**	**47.14 mg**	**Delphinidin 3-O-arabinoside**	**13.98 mg**	Pelargonidin 3-O-glucoside	1.65 mg				
Petunidin 3-O-rutinoside	4.09 mg	Pelargonidin 3-O-rutinoside	1.32 mg	**Delphinidin 3-O-galactoside**	**20.50 mg**	Pelargonidin 3-O-glucosyl-rutinoside	0.82 mg				
				Delphinidin 3-O-glucoside	8.08 mg	Pelargonidin 3-O-rutinoside	0.42 mg				
				Malvidin 3-O-(6''-acetyl-galactoside)	2.64 mg	Pelargonidin 3-O-sophoroside	3.46 mg				
				Malvidin 3-O-(6''-acetyl-glucoside)	3.29 mg						
				**Malvidin 3-O-arabinoside**	**13.42 mg**						
				**Malvidin 3-O-galactoside**	**17.23 mg**						
				**Malvidin 3-O-glucoside**	**11.18 mg**						
				Peonidin 3-O-(6''-acetyl-galactoside)	0.03 mg						
				Peonidin 3-O-(6''-acetyl-glucoside)	0.39 mg						
				Peonidin 3-O-arabinoside	0.29 mg						
				Peonidin 3-O-galactoside	0.90 mg						
				Peonidin 3-O-glucoside	0.20 mg						
				Petunidin 3-O-(6''-acetyl-galactoside)	0.06 mg						
				Petunidin 3-O-(6''-acetyl-glucoside)	1.35 mg						
				Petunidin 3-O-arabinoside	8.53 mg						
				**Petunidin 3-O-galactoside**	**12.73 mg**						
				Petunidin 3-O-glucoside	6.09 mg						

Blackcurrant		Strawberry		Blueberry		Raspberry		Blackberry		Cranberry	
Flavanols											
(+)-Catechin	0.70 mg	(+)-Catechin	6.36 mg	(-)-Epicatechin	1.11 mg	(+)-Catechin	0.58 mg	(+)-Catechin	0.72 mg		
(-)-Epicatechin	0.47 mg	(+)-Gallocatechin	0.05 mg			(-)-Epicatechin	5.05 mg	(-)-Epicatechin	11.48 mg		
		(-)-Epicatechin	7.5e-03 mg			Procyanidin dimer B2	0.10 mg	(-)-Epigallocatechin	0.15 mg		
		(-)-Epicatechin 3-O-gallate	0.28 mg					Procyanidin dimer B1	0.23 mg		
		(-)-Epigallocatechin	0.06 mg					Procyanidin dimer B2	0.83 mg		
		Procyanidin dimer B1	0.62 mg					Procyanidin dimer B3	0.33 mg		
		Procyanidin dimer B2	0.03 mg					Prodelphinidin dimer B3	0.07 mg		
		Procyanidin dimer B3	1.10 mg					Procyanidin trimer EEC	0.06 mg		
		Procyanidin dimer B4	0.13 mg								
		Procyanidin trimer EEC	0.50 mg								
Blackcurrant		Strawberry		Blueberry		Raspberry		Blackberry		Cranberry	
Flavonols											
Kaempferol 3-O-glucoside	0.57 mg	Kaempferol 3-O-glucoside	0.32 mg	Kaempferol 3-O-glucoside	0.62 mg	Kaempferol	2.14e-03 mg	Quercetin 3-O-galactoside	4.10 mg	Kaempferol 3-O-glucoside	0.87 mg
Myricetin 3-O-glucoside	2.71 mg	Kaempferol 3-O-glucuronide	0.20 mg	Myricetin 3-O-arabinoside	12.21 mg	Kaempferol 3-O-glucoside	1.03 mg	Quercetin 3-O-glucoside	0.67 mg	Myricetin 3-O-arabinoside	5.30 mg
Myricetin 3-O-rutinoside	3.14 mg	Morin	0.06 mg	Myricetin 3-O-rhamnoside	1.03 mg	Quercetin	0.02 mg	Quercetin 3-O-glucosyl-xyloside	0.46 mg	Quercetin 3-O-arabinoside	4.94 mg
Quercetin 3-O-glucoside	2.61 mg	Quercetin 3-O-glucuronide	1.74 mg	Quercetin 3-O-acetyl-rhamnoside	5.66 mg	Quercetin 3-O-glucoside	3.58 mg	Quercetin 3-O-rutinoside	3.89 mg	Quercetin 3-O-galactoside	10.81 mg
Quercetin 3-O-rutinoside	4.65 mg			Quercetin 3-O-arabinoside	7.09 mg	Quercetin 3-O-glucuronide	0.63 mg	Quercetin 3-O-xyloside	1.28 mg	Quercetin 3-O-rhamnoside	6.17 mg
				Quercetin 3-O-galactoside	8.99 mg	Quercetin 3-O-rutinoside	11.00 mg	Quercetin 3-O-xylosyl-glucuronide	2.37 mg		
				Quercetin 3-O-glucoside	1.49 mg						
				Quercetin 3-O-xyloside	1.60 mg						
Blackcurrant		Strawberry		Blueberry		Raspberry		Blackberry		Cranberry	
Phenolic acids											
Hydroxybenzoic acids											
4-Hydroxybenzoic acid 4-O-glucoside	0.73 mg	4-Hydroxybenzoic acid 4-O-glucoside	1.53 mg	4-Hydroxybenzoic acid 4-O-glucoside	0.55 mg	**Ellagic acid**	**48-164***	4-Hydroxybenzoic acid 4-O-glucoside	1.13 mg	2,4-Dihydroxybenzoic acid	0.80 mg
5-O-Galloylquinic acid	0.07 mg	5-O-Galloylquinic acid	0.05 mg	Gallic acid 4-O-glucoside	0.50 mg	Ellagic acid acetyl-arabinoside	0.20 mg	**Ellagic acid**	**45-84***	3-Hydroxybenzoic acid	0.41 mg
Gallic acid 4-O-glucoside	0.10 mg	Ellagic acid	4.9–97.8*	Protocatechuic acid 4-O-glucoside	0.40 mg	Ellagic acid acetyl-xyloside	0.36 mg	Gallic acid	4.67 mg	4-Hydroxybenzoic acid	0.42 mg
Galloyl glucose	0.53 mg	Ellagic acid glucoside	2.85 mg			Ellagic acid arabinoside	2.27 mg	Galloyl glucose	0.27 mg	**Benzoic acid**	**48.10 mg**
Protocatechuic acid 4-O-glucoside	0.07 mg	**Agrimoniin**	**25.0–747***			**Lambertianin C**	**285-627***	Protocatechuic acid 4-O-glucoside	0.43 mg	Vanillic acid	2.81 mg
		**Casuarictin**	**19.0–386***			**Sanguiin H-6**	**367-742***	**Lambertianin C**	**315-665***		
		**Sanguiin H6 isomer**	**1.5–133***					**Sanguiin H-6**	**189-418***		
Hydroxycinnamic acids											
3-Caffeoylquinic acid	4.30 mg	5-Caffeoylquinic acid	1.93 mg	3-Caffeoylquinic acid	0.60 mg	5-Caffeoylquinic acid	0.57 mg	3-Caffeoylquinic acid	4.53 mg	Caffeic acid	2.31 mg

3-Feruloylquinic acid	0.07 mg	Caffeoyl glucose	0.10 mg	4-Caffeoylquinic acid	0.35 mg	p-Coumaric acid	2.30e-04 mg	3-Feruloylquinic acid	0.30 mg	Cinnamic acid	0.16 mg
3-p-Coumaroylquinic acid	1.73 mg	Cinnamic acid	0.22 mg	**5-Caffeoylquinic acid**	**131.2 mg**	p-Coumaric acid 4-O-glucoside	0.32 mg	3-p-Coumaroylquinic acid	0.37 mg	Ferulic acid	0.81 mg
4-Caffeoylquinic acid	0.37 mg	Feruloyl glucose	0.10 mg	5-Feruloylquinic acid	0.75 mg			4-Caffeoylquinic acid	0.10 mg	p-Coumaric acid	1.08 mg
4-Feruloylquinic acid	0.23 mg	p-Coumaric acid	0.21 mg	5-p-Coumaroylquinic acid	0.35 mg			5-Caffeoylquinic acid	0.10 mg		
4-p-Coumaroylquinic acid	0.13 mg	p-Coumaric acid 4-O-glucoside	0.15 mg	Caffeic acid 4-O-glucoside	0.30 mg			Caffeoyl glucose	0.50 mg		
5-Caffeoylquinic acid	0.13 mg	p-Coumaroyl glucose	4.36 mg	Ferulic acid 4-O-glucoside	0.55 mg			Feruloyl glucose	0.43 mg		
5-Feruloylquinic acid	0.13 mg	Resveratrol	0.35 mg	p-Coumaric acid 4-O-glucoside	0.95 mg			p-Coumaric acid 4-O-glucoside	0.27 mg		
Caffeic acid 4-O-glucoside	0.20 mg							p-Coumaroyl glucose	0.67 mg		
Caffeoyl glucose	2.79 mg										
Ferulic acid 4-O-glucoside	0.27 mg										
Feruloyl glucose	1.30 mg										
p-Coumaric acid 4-O-glucoside	0.51 mg										
p-Coumaroyl glucose	1.13 mg										
**Blackcurrant**		**Strawberry**		**Blueberry**		**Raspberry**		**Blackberry**		**Cranberry**	
**Aroma Compounds**											
**3-carene**		**Furaneol**		6-methyl-5-hepten-2-one		**4-(p-Hydroxyphenyl)-2-butanone**		**5-hydroxymethylfurfural**		**Ursolic acid**	
**Humulene**		**Mesifurane**		p-Cymen-8-ol		benzyl alcohol		**2,3-dihydro-3,5-dihydroxy-6-methyl -4H-pyran-4-one**		**10-p-*trans* - coumaroyl-1S-dihydromonotropein**	
**Sabinene**		Methional		E-2-Hexen-1-ol		(Z)-3-hexen-1-ol		**2-heptanol**		**10-p-*cis* -coumaroyl-1S-dihydromonotropein**	
**Terpinolene**		Methyl anthranilate		E-3-Hexen-1-ol		*Alfa* -pinene		6-methyl-5-hepten-2-ol		**cis-3-O-p-hydroxycinnamoyl ursolic acid**	
Beta -Damascenone		Farnesyl acetate		Hexanal		*Beta* -pinene		camphene		**trans-3-O-p-hydroxycinnamoyl ursolic acid**	
Furfural		Linalool		E-2-Hexenal		*Alfa* -phellandrene		*alfa* -terpineol		**2-O-(3,4-dihydroxybenzoyl)-2,4,6- Trihydroxyphenyl methylacetate**	
cis -3-Hexen-1-ol		Geraniol		Butyrolactone		Linalool		2-undecanone		Cinnamyl alcohol	
Beta -phellandrene		Gamma-Decalactone		Limonene		Geraniol		Ethyl-2-methylpropanoate		2-phenyl ethanol	
Beta -cymenene		Gamma-Dodecalactone		Nerol		Citral		Ethyl butanoate		1-phenyl ethanol	
Beta -ocimene		2-heptanone		Linalool		*Alfa* -Caryophyllene		Methyl-2-methylbutanoate		Cinnamaldehyde	
Germacrene		1-Octen-3-one		Geraniol		*Alfa* -ionone		Ethyl hexanoate		Phenylacetaldehyde	
Methyl butanoate		Hexanal		Terpineol		*Beta* -ionone		Hexanal			
Ethyl butanoate		(E)-2-Hexenal				6-methyl-5-hepten-2-one		E-2-Hexenal			
Ethyl hexanoate						2-heptanol		2-Heptanone			
Nonanal						terpinen-4-ol		Alfa-pinene			
Alfa -pinene						edulan		linalool			
2,3-Butanedione								geraniol			
1-Octen-3-one								Myrtenol			
1,8-Cineole								Damascenone			
Linalool								*Alfa* -ionone			
Terpinen-4-ol								*Beta* -ionone			
Alfa -terpineol								Furaneol			

Beta -Damascenone				Methional	
2-methylbutyl acetate				p-Cymen-8-ol	
4-Methoxy-2-methylbutanethiol					
methyl-2methyl butanoate					

1.Blumberg JB, Camesano TA, Cassidy A, et al (2013) Cranberries and Their Bioactive Constituents in Human Health. Adv Nutr 4:618–632. https://doi.org/10.3945/an.113.004473

2.Boccorh RK, Paterson A, Piggott JR (2002) Extraction of aroma components to quantify overall sensory character in a processed blackcurrant (Ribes nigrum L.) concentrate. Flavour Fragr J 17:385–391. https://doi.org/10.1002/ffj.1111

3.Brown PN, Murch SJ, Shipley P (2012) Phytochemical Diversity of Cranberry (Vaccinium macrocarpon Aiton) Cultivars by Anthocyanin Determination and Metabolomic Profiling with Chemometric Analysis. J Agric Food Chem 60:261–271. https://doi.org/10.1021/jf2033335

4.Đorđević BS, Pljevljakušić DS, Šavikin KP, et al (2014) Essential Oil from Blackcurrant Buds as Chemotaxonomy Marker and Antimicrobial Agent. Chem Biodivers 11:1228–1240. https://doi.org/10.1002/cbdv.201400039

5.El Hadi M, Zhang F-J, Wu F-F, et al (2013) Advances in Fruit Aroma Volatile Research. Molecules 18:8200–8229. https://doi.org/10.3390/molecules18078200

6.Filly A, Fabiano-Tixier A-S, Lemasson Y, et al (2014) Extraction of aroma compounds in blackcurrant buds by alternative solvents: Theoretical and experimental solubility study. Comptes Rendus Chim 17:1268–1275. https://doi.org/10.1016/j.crci.2014.03.013

7.Gasperotti M, Masuero D, Guella G, et al (2013) Evolution of Ellagitannin Content and Profile during Fruit Ripening in Fragaria spp. J Agric Food Chem 61:8597–8607. https://doi.org/10.1021/jf402706h

8.Gasperotti M, Masuero D, Vrhovsek U, et al (2010) Profiling and Accurate Quantification of Rubus Ellagitannins and Ellagic Acid Conjugates Using Direct UPLC-Q-TOF HDMS and HPLC-DAD Analysis. J Agric Food Chem 58:4602–4616. https://doi.org/10.1021/jf904543w

9.Grochowski DM, Skalicka-Woźniak K, Orhan IE, et al (2017) A comprehensive review of agrimoniin. Ann N Y Acad Sci 1401:166–180. https://doi.org/10.1111/nyas.13421

10.Turemis K, Kafkas E, Kafkas S, et al (2003) Determination of Aroma Compounds in Blackberry by GC/MS Analysis. Chem Nat Compd 39:. https://doi.org/10.1023/A:1024809813305

11.Vrhovsek U, Guella G, Gasperotti M, et al (2012) Clarifying the Identity of the Main Ellagitannin in the Fruit of the Strawberry, Fragaria vesca and Fragaria ananassa Duch. J Agric Food Chem 60:2507–2516. https://doi.org/10.1021/jf2052256

12.http://phytohub.eu/
